# Effect of Annealing on Direct Recycled NMC Cathodes

**DOI:** 10.1021/acs.chemmater.5c01824

**Published:** 2025-11-24

**Authors:** Juliane I. Preimesberger, Cyrus K. Kirwa, Eva Allen, Nikita S. Dutta, Evelyna Wang, Fulya Dogan, Patrick Walker, Yaocai Bai, Krzysztof Z. Pupek, Lisa Stanley, Tiffany L. Kinnibrugh, Matthew Nisbet, Timothy T. Fister, Hongmei Luo, Jaclyn E. Coyle

**Affiliations:** † Materials, Chemical, and Computational Science Center, 53405 National Renewable Energy Laboratory, Golden, Colorado 80401, United States; ‡ Department of Chemical and Materials Engineering, 4423 New Mexico State University, Las Cruces, New Mexico 88003, United States; § Chemical Sciences and Engineering Division, 1291 Argonne National Laboratory, Lemont, Illinois 60439, United States; ∥ Electrification and Energy Infrastructures Division, 6146 Oak Ridge National Laboratory, Oak Ridge, Tennessee 37830, United States; ⊥ Applied Materials Division, Argonne National Laboratory, Lemont, Illinois 60439, United States

## Abstract

A substantial number of electric vehicle batteries are poised to reach end-of-life conditions in the next decade. Direct recycling has advantages over typical recycling processes because it preserves the chemical structure of the material. One key step of direct recycling materials like nickel manganese cobalt oxide (NMC) cathodes is relithiation, which includes replacing the depleted lithium inventory in the cathode and annealing the material to fix crystallographic degradation. This work uses multiple characterization techniques (synchrotron X-ray diffraction, Ni X-ray absorption near edge structure, scanning transmission electron microscopy) to understand the lithiation mechanism of degraded and chemically relithiated NMC 622. Despite the necessary reconstruction after relithiation being limited to the surface of the degraded NMC 622, a high annealing temperature of 720 °C is still necessary to restore the NMC 622 structure back to pristine condition after relithiation. This work also finds that treating degraded NMC 622 with an annealing step is sufficient to restore key electrochemical and structural properties, other essential features of pristine NMC 622 material such as particle porosity and morphology are largely unaffected. Understanding the effect of this annealing step has important implications on defining the degree of success of any given direct recycling strategy.

## Graphical abstract

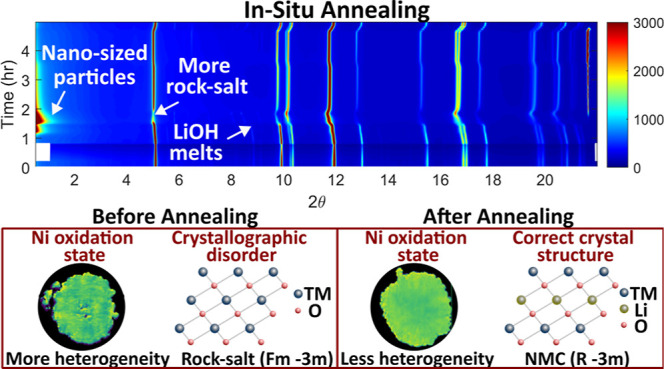

## Introduction

In 2023, an estimated one-in-five cars sold worldwide were electric vehicles, and projected trends indicate that electric vehicles’ (EVs) market share could increase substantially by 2035.^1^ This means a significant amount of EV batteries will reach end-of-life in the next decade. Lithium-ion batteries (LIBs) that are too degraded to be refurbished or reused in other applications (such as stationary storage) must either be recycled or sent to the landfill. Given the value of the materials in current LIBs, there is considerable incentive to recover end-of-life (EoL) materials via recycling.

Commercial-scale recycling of LIBs mostly uses either hydrometallurgical or pyrothermal processes, which leach or burn LIBs to recover valuable metal components.^2^ However, these processes produce substantial emissions and toxic waste streams. A promising alternative, called direct recycling, is able to recover LIB materials without changing the chemical structure of the material, and typically does not produce as much emissions as hydro- or pyrothermal recycling.^2–4^ Most direct recycling studies, demonstrated at lab and pilot scales, have focused on recycling LIB cathodes, which have valuable metals like lithium, cobalt, manganese, and nickel, and a complicated, carefully engineered crystal structure that was time- and cost-intensive to manufacture.^5,6^ Thus, recovering the cathode material without damaging its structure results in an output that is higher value than just the cost of the metals alone.

An ideal direct recycling process would address the major degradation mechanisms that typically plague cathode materials: loss of lithium inventory, crystallographic changes, and particle fracture.^7^ One direct recycling method for cathode materials is relithiation, which replenishes the lithium lost due to solid-electrolyte-interphase (SEI) formation and other capacity fade mechanisms.^8,9^ While there are many methods for relithiation, one particularly promising one uses a redox mediator to act as a charge shuttle that delivers lithium ions and electrons to a lithium-deficient cathode.^8,9^ This relithiation technique works at room temperature, is fast, and does not require knowledge of the amount of lithium deficiency in the EoL material.^8,9^

After this relithiation step, a high-temperature annealing step follows, which ideally converts the rock-salt type crystal structure formed due to local lithium deficiencies during battery usage or relithiation into the correct layered oxide structure.^10^ This annealing step is crucial to the success of relithiation processes and can be quite expensive as high amounts of energy are needed for a high-temperature furnace. The typical synthesis temperature starting from precursor cathode materials is 850 °C, but it may be possible to use a lower temperature for annealing the recycled materials, which could save recycling costs.^10^ Previous work has demonstrated successful relithiation of EoL nickel manganese cobalt oxide (NMC) cathode materials and shown that a lower annealing temperature of 720 °C at 8 h with an additional source of lithium mixed into the material is crucial to obtain recycled material that replicates pristine material electrochemical performance.^9^ However, the mechanism behind a successful annealing step is yet to be fully understood.

There have been numerous X-ray diffraction studies on the synthesis and degradation during battery operation of NMC cathode materials, because they reveal important structural information like the quality of the oxide layering and the amount of cation mixing, where nickel ions switch into a lithium site in the layered oxide lattice.^10–13^ Synchrotron diffraction studies are particularly useful, because the rock-salt crystal structure is typically a surface layer, and may not show up in the typical, lower intensity laboratory X-ray diffraction measurements.^10^ Studies investigating the crystal structure of degraded or rejuvenated cathode materials are fewer, however, and these studies have not focused on how the cathode chemical structure changes during high-temperature annealing steps after relithiation.^3,4,14^

In this study, we try to understand the mechanism of lithiation during annealing for a specific case study where the prior chemical relithiation likely leaves lithium at the surface and may not uniformly relithiate the material without the annealing step. For cathode synthesis, the annealing step is controlled to determine crystallinity, internal porosity, and primary and secondary particle size.^10,15,16^ However, with end-of-life and recycled materials, it is unclear how already degraded cathode material is affected by high temperatures: does annealing worsen crystal defects or fix them? This study aims to determine what a “successful” relithiation annealing step would be and what features of a commercial EoL material can be preserved or recovered during annealing.

Battery packs that will be EoL in the next five to ten years will likely have higher nickel content, because battery manufacturers have been moving to higher nickel content cathodes to lower cost by limiting cobalt usage.^6,17^ As such, this paper focuses on EoL and relithiated NMC 622 (Ni/Mn/Co 6:2:2). EoL NMC 622 material was harvested from an electric vehicle battery, put through the redox mediator relithiation process, and then characterized during the annealing step. We use synchrotron diffraction, transmission X-ray microscopy (TXM) with Ni X-ray absorption near edge structure (XANES), scanning transmission electron microscopy (STEM), and electrochemical characterization to understand how the crystal structure of the material changes during the annealing step. We confirm that an additional lithium source, in the form of a lithium salt, is crucial for proper annealing of the recycled cathode material, and that a lower temperature annealing step of 720 °C is sufficient to correct crystallographic degradation and recover pristine electrochemical performance. We also find that annealing could affect NMC particle structure such as internal porosity and primary particle size.

## Experimental Section

### Relithiation Procedure

EoL NMC 622 cathode material was harvested from a commercial electric vehicle battery pack (80% capacity retention from pristine). Cells were shredded, electrodes were sorted by hand and put through a solvent separation process using triethyl phosphate (TEP) and acetone to separate out the cathode material from the aluminum current collector.^18^ The EoL material was sieved to remove clumps and washed with 0.5 M LiOH in deionized water solution to remove surface impurities (all EoL material in this paper underwent this washing step prior to characterization). The washed EoL material was then relithiated using a redox mediator process.^8^ A 1 M solution of redox mediator (RM) 2,5-di-*tert*-butylhydroquinone (DTBHQ) in 1,2-dimethoxyethane (DME), with 4 M LiOH added, was stirred overnight in an argon-filled glovebox. Five g of the EoL material was then added to this solution and stirred for 2 h at 60 °C to relithiate it. The material is then recovered via filtering, rinsed with DME, and dried at 80 °C overnight under vacuum. Further details about the impact of the washing step, the selection of redox mediators, and how this affects material surface chemistry and electrochemical performance, are discussed in Kirwa et al.^9^

A full annealing step consists of mixing 1.8 wt % of LiOH into 5 g of the relithiated material and heating the sample in air to 720 °C for 8 h. Electrochemical tests were performed on samples that were annealed with and without LiOH, for 4 and 8 h. Synchrotron diffraction samples were annealed at two temperatures, 720 and 850 °C.

### Electrochemical Characterization

Cathodes were made with 8:1:1 w/w/w NMC/PVDF­(Alfa Aesar)/Carbon black (Timcal Super C65) ratios, in a *N*-methyl-2-pyrrolidone (Sigma-Aldrich) solvent. The cathode slurry was cast onto aluminum foils with a wet gap of 150 μm and then dried at 150 °C for 4 h under vacuum. Coin cells in the 2032-type configuration were built with a 14 mm cathode punch against lithium metal (9/16″ diameter, Alfa Aesar 10,769, 0.75 mm thick, 99.9% metals basis), with 50 μL Gen2 electrolyte (1.2 M lithium hexafluorophosphate LiPF_6_ in EC/EMC 3:7 w/w). Cells were cycled on a Maccor Series 4000 battery tester system in an temperature-controlled oven at 30 °C. Half cells were cycled at a C/10 rate from 3.0 to 4.3 V vs Li/Li^+^.

### In-Situ Synchrotron Annealing

Powder diffraction during the upcycling process was measured at the adpanced photon source, sector 13BM-C. Measurements used the higher energy, fixed-angle 311 side bounce monochromator at 28.6 keV (λ = 0.4255 Å) with a focused beam (150 × 185 μm^2^). Powder diffraction was measured using a Pilatus3 1 M detector located 450 mm behind the sample and was calibrated using a LaB_6_ NIST standard. As shown in Figure 3a, a few mg of the starting material was loaded in 1 mm sapphire capillaries that were backfilled with dry air and terminated with alumina frits to allow for gas diffusion. The samples were heated by a nitrogen-jet furnace developed at APS for these measurements and were monitored and controlled using a thermocouple located just below the samples, seen in the inset of Figure 3a. During the thermal ramp, the three samples were measured by rotating between each sample tube, staying within the heating zone defined by the inner diameter of the ceramic heater. Each 15 s diffraction measurement was also conducted while rotating the individual capillary by 10° for powder averaging.

Three samples were annealed simultaneously: the washed EoL material, the relithiated EoL material, and relithiated EoL material with 1.8 wt % LiOH added. There were two temperature holds: 720 °C (held for 1 h 25 min) and 850 °C (held for 1 h), and temperature ramps at 500 °C/h, followed by a rapid cool-down. Data was binned into scans of approximately 225 s, and Rietveld refinement was performed using TOPAS (v6) software.

### TXM and Ni XANES

Transmission X-ray microscopy (TXM) was conducted at the full-field X-ray imaging (FXI) beamline (18-ID) at Brookhaven National Laboratory (BNL), National Synchrotron Light Source II (NSLS-II). The cathode material was deagglomerated using a 45 μm sieve and packed into a quartz capillary with an inner diameter of 50 μm and an outer diameter of 80 μm. An angular range between 0° and 180° was used to collect X-ray projection images and reconstructed into 3D tomograms using the Gridrec algorithm within the Tomopy package through the “FXI tomo util” interface developed at FXI.^19^ TXM X-ray absorption near edge spectroscopy (XANES) was captured of the Ni K-edge by collecting tomograms with a total of 63 energies between 8.15 and 8.9 keV. Each tomogram was aligned and the Ni valence state was calculated by the white line position referenced to samples of LiNiO_2_ and NiO using the pyXAS package developed at FXI.^20^ The Ni valence state trends were confirmed by mapping the relative Ni peak position shown in Figure S8. Additional 25 energies were collected across the Mn (6.0–6.96 keV) and Co (7.0–8.16 keV) edges to generate maps of the relative elemental concentration by calculating the difference in absorption between pre- and postedges.

All data rendering and quantification were conducted in the ORS Dragonfly Software.^21^ The method developed by Allen; et al. *ACS Energy Lett.* 2023 was used to generate the depth-dependent measurements of elemental content and oxidation state.^22^ First, the particles were segmented using a combination of Otsu thresholding, morphological-based segmentation, and itemization. Individual particle surfaces were calculated and used as a reference point to generate Euclidean distance maps. The mean values of the map intensity as a function of Euclidean map position with the 99% confidence intervals were plotted for five individual particles and all particles within the field-of-view (FOV) collectively.

### Other Materials Characterization

Electrode cross-sectional samples for scanning transmission electron microscopy (STEM) were prepared by Xe plasma beam milling using a Thermo Fisher Helios Hydra plasma focused ion beam (PFIB). STEM imaging was performed on an aberration-corrected Thermo Fisher Spectra 200 S/TEM, operated at 200 kV with a 24.2 mrad convergence angle and the screen current maintained at <50 pA throughout imaging.

Particle size was measured with a Bettersize S3 Plus. Thermogravimetric analysis (TGA–FTIR) evolved gas analysis was performed on EoL, relithiated, and annealed powder samples (∼10 mg loaded in an alumina crucible) using a TA Instruments Discovery SDT 650 instrument connected to a PerkinElmer FT-IR Spectrum 3 spectrometer equipped with a TL 8000 Balanced Flow FT-IR EGA System, with a PerkinElmer TGA–IR Interface TL 8000e controlling the temperature of the adapter, cell and TL–TGA to 270 °C, and the flow rate to 70 mL/min. Mass loss, measured under nitrogen at a flow rate of 100 mL/min, was measured up to 900 °C with a ramp rate of 50 °C/min. To analyze the evolved gas, FT-IR measurements were taken from 650 to 4000 cm^–1^, with a resolution of 4 cm^–1^ and an accumulation of two scans. A background spectrum was performed on the spectrum 3 before each sample was run with a resolution of 4 cm^–1^ and an accumulation of 64 scans. All TGA curves were analyzed using TA Instruments Trios software while all FT-IR spectra were analyzed using PerkinElmer spectrum 10 IR software.

Solid-state magic-angle-spinning (MAS) nuclear magnetic resonance (NMR) was performed on a 300 MHz (7.04 T) Bruker AVANCE III spectrometer using a 1.3 mm HXY WB MAS probe. Powder samples were loaded into 1.3 mm zirconia rotors in an argon-filled glovebox. Solid ^6,7^Li MAS NMR spectra were acquired using a rotor-synchronized hahn echo experiment at 283 K with at a 60 kHz rotation rate and recycle delays of 0.2 (^6^Li) and 15 (^7^Li) seconds.

## Results and Discussion

NMC 622 pristine (Targray), EoL and recycled samples were tested in coin half cells for electrochemical performance. For more electrochemical testing, see Kirwa et al., where the recycled cathode material was shown to replicate pristine NMC 622 results in both rate capability and full cell cycle lifetime.^9^ The first charge–discharge cycle is shown in Figure 1a, and the first and reversible capacities are shown in Figure 1b. Recycled samples were annealed after redox-mediated relithiation at 720 °C, for 4 or 8 h, with or without 7 mol % (or 1.8 wt %) LiOH mixed into the relithiated material. Previous studies report lithium evaporation during annealing, which leads to cation mixing and the formation of the rock-salt (RS) phase, so it is crucial to provide enough lithium to overcome this evaporation.^12^ From Figure 1b, it is evident that the EoL material has lost significant capacity compared to the pristine material. While the battery pack that the EoL material was harvested from was at 80% capacity retention, the EoL material has more significant capacity loss, possibly due to higher degradation of the cathode in the pack, or damages from upstream recycling processes. Redox-mediated relithiation and annealing restores the capacity of the cathode material. Adding additional LiOH during annealing does help improve recycled capacities, but the major difference is annealing time. It appears that 4 h is not sufficient to fully fix the crystal structure. An annealing step, which anneals at 720 °C for 8 h with extra LiOH is able to convert the NMC material back to pristine values, indicating that the lower temperature annealing hold is sufficient to fully recycle the material.

**Figure 1:**
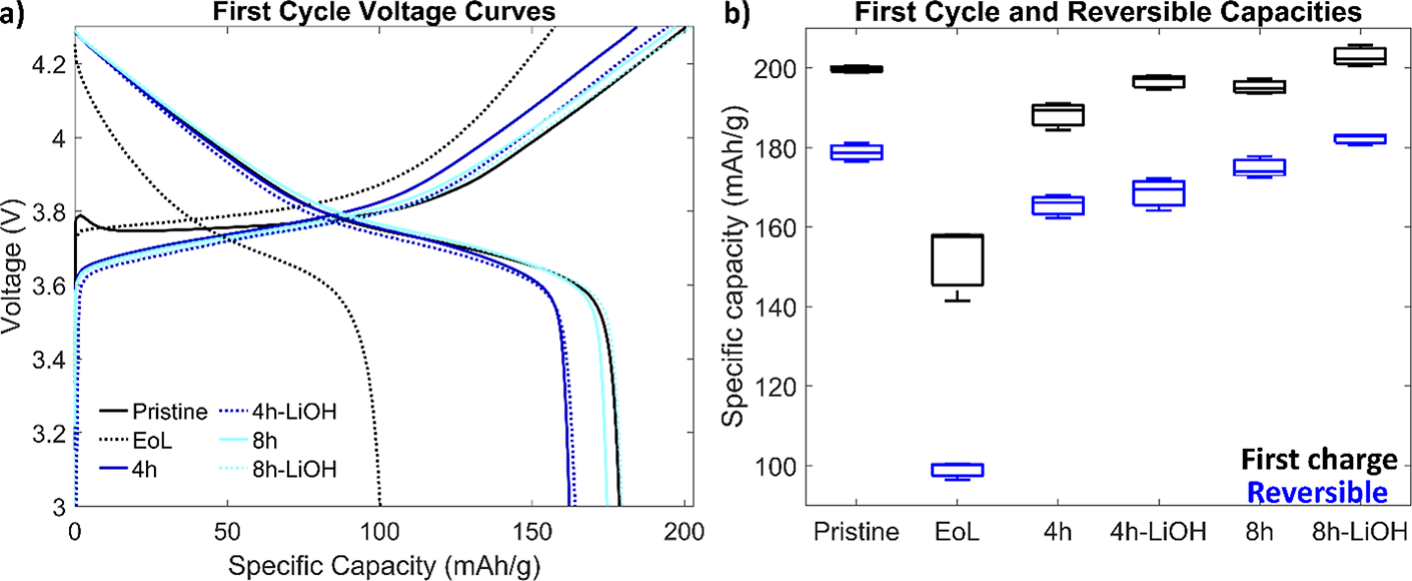
Electrochemical results. (a) Half-cell first cycle charge–discharge curves for various NMC cathodes. (b) First cycle and reversible capacities from three replicate cells.

To investigate the NMC 622 structure at different stages of the recycling process, STEM characterization was performed on pristine, EoL, relithiated, and annealed electrode samples. In this paper, samples called “relithiated” have gone through the redox relithiation process but have not been annealed, and samples called “annealed” have been annealed at 720 °C with 1.8 wt % LiOH for 8 h after redox relithiation, unless stated otherwise. STEM reveals that the samples primarily had a layered structure, with a disordered surface layer, as shown in Figure 2a (see Figure S1 for images of all samples). The layered structure is the trigonal *R-3mH* layered oxide structure (referred to in this paper as the NMC phase). The disordered surface includes areas of a cubic *Fm*3̅*m* lattice that matches a rock-salt (RS) structure (shown in Figure 2b). This RS phase is typically found in the NMC 622 material during synthesis due to local lithium vacancies but is converted back into the NMC structure once high enough temperatures are reached.^10,12^ This RS structure also appears in degraded NMC material and contributes to cathode capacity loss; however, even pristine cathode material will still have a small amount of this rock-salt phase.^4,12^

**Figure 2:**
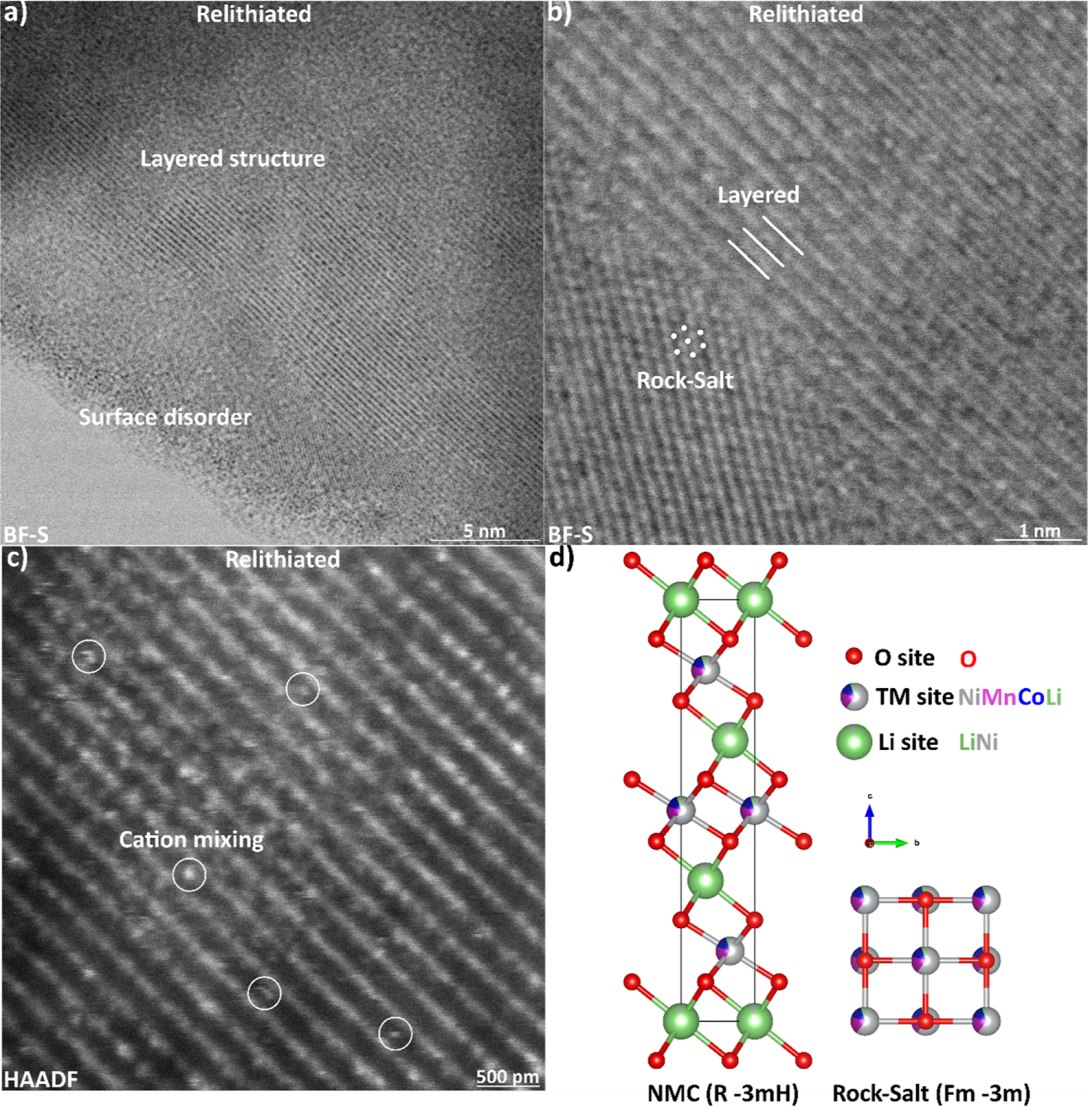
Crystal structures found in relithiated cathode materials. (a) Bright-field (BF) STEM image of the surface disorder present in a relithiated sample. (b) BF STEM image of cubic rock-salt structure next to the layered oxide structure. (c) HAADF–STEM image of cation mixing (circled) visible as bright spots in the dark lithium layer. (d) Crystal structures of the layered oxide phase (NMC) and the rock-salt phase (RS).

Cation mixing (i.e., when the Ni^3+^ ion is reduced to a Ni^2+^ ion and switches from the transition metal (TM) site into the lithium site in the NMC layered oxide phase) is known to occur in these materials.^4,10–13,23^ Generally, a lower degree of cation mixing is more structurally stable, and higher amounts of cation mixing occur in degraded cathodes.^4,23^ Shown in Figure 2c is a high-angle annular dark field (HAADF) STEM image, where bright rows indicate the transition metal atom layers, and the dark lines are the lithium layers. Cation mixing is visible as bright spots (TM atoms) in the dark lithium layer, as marked in Figure 2c. The crystal structures of the NMC and RS phases are shown in Figure 2d, drawn using VESTA 3.^24^ For both structures, lithium ions can move into the transition metal (TM) sites. While STEM shows examples of cation mixing, the sample size is too small for quantitative measurements.

Therefore, to study the NMC 622 crystal structure during the annealing step, we use in situ synchrotron diffraction, which has higher intensity X-rays than laboratory X-ray diffraction tools, enabling measurement of cation mixing and the surface RS phase. Figure 3a is an image of the beamline setup, with the beam direction and 2θ rotation annotated. The inset shows the thermocouple that controls the furnace. Figure 3b shows the in situ heating profile for the annealing step, with two temperature holds at 720 and 850 °C. The 720 °C temperature was chosen as this is consistent with good electrochemical results in the annealed material, as shown in Figure 1. This temperature was demonstrated as a successful annealing temperature in a previous work, and is within the typical calcination temperatures of high nickel content NMC materials.^9,23^ The 850 °C temperature was chosen for comparison as this is consistent with typical NMC synthesis from precursors.^10^ Due to limitations in beam time, only hour-long holds at each temperature were performed, though a typical annealing step would require 8 h. The shorter hold time is still expected to be representative of structural changes at these temperatures. Synchrotron diffraction patterns were taken continuously and binned into scans of approximately 225 s for Rietveld refinement. The full diffraction patterns are shown in Figure S2. The χ^2^ values, calculated as the square of TOPAS’ goodness of fit parameter, (shown in Figure 3c) indicate a good quality fit (all χ^2^ < 5, with most scans χ^2^ < 3). The residuals of each fit are shown in Figure S3. Figure 3d shows the Rietveld refinements and errors for each of the samples at room temperature.

**Figure 3:**
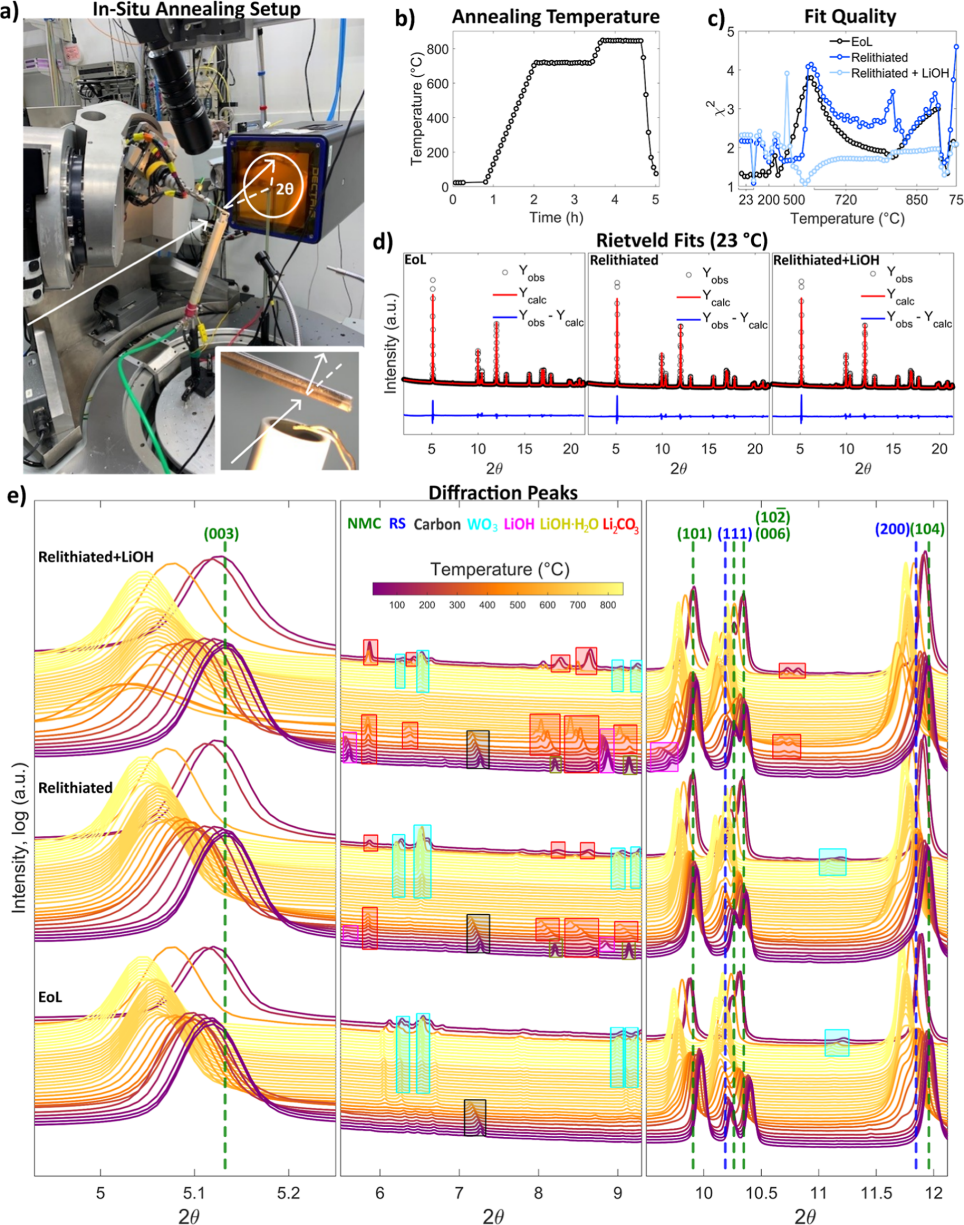
Diffraction patterns and Rietveld fits. (a) Image of experimental setup. (b) Annealing temperature profile versus time. (c) Fit quality (χ^2^) of Rietveld refinements. (d) Rietveld refinements and error for each powder at room temperature; Rietveld fit was fit to a 2θ range of 2–21.4, to exclude small-angle scattering and an artifact peak in the relithiated + LiOH sample at 21.5. (e) Important peaks from the diffraction patterns during annealing for each sample. NMC and RS peaks are labeled with *hkl* values, and significant peaks from other phases are shaded. Every other diffraction pattern is shown for better clarity.

A major contribution to spikes in the χ^2^ value is due to the in situ heating, which means a single scan is an average of a range of temperatures. There could also be local inhomogeneity in the structures due to kinetic limitations during heating. Other imperfections in the fit might be due to another intermediate phase, which has been noted in other XRD studies looking at NMC EoL material.^4^ One possible structure for this phase is a monoclinic structure, which is known to appear in lithium cobalt oxide (LCO) and NMC cathode materials due to local lithium vacancies.^25–28^ While fitting this structure does result in slight improvements in the χ^2^ value, this phase completely overlaps with other phases’ peaks and there are limited studies of a monoclinic phase for NMC 622 (see Supporting Information Figure S4 and Table S1 for further discussion). Therefore, we do not include this structure in the Rietveld refinements, because attempts to fit this phase do not change the lattice parameters or cation mixing amounts of the NMC phase significantly and proposing a new structure is left for future study.

Figure 3e shows the diffraction patterns of prominent peaks for all powders throughout annealing, with locations of significant peaks for all phases marked, and the *hkl* values of the NMC and RS phases labeled. The dashed lines for the NMC and RS peaks indicate where the peak location is for the relithiated + LiOH sample after annealing and cooling down (at ∼75 °C). The NMC (003) peak for all samples shifts to lower angles due to thermal expansion during annealing, as expected. The (003) peak of both relithiated materials at room temperature is shifted to higher angles compared to the EoL material, consistent with a successful relithiation, as lithium insertion contracts the *c*-axis of the NMC lattice. At around 500 °C, the relithiated + LiOH (003) peak has significant peak broadening. Wider peaks in X-ray diffraction could indicate smaller crystallite sizes or more strain in the lattice.^29^ This peak broadening only shows up in the relithiated + LiOH sample, suggesting a fundamentally different mechanism for this material during annealing. Also at this temperature, a large portion of the material converts from the layered-oxide NMC phase to the cubic RS phase for all three samples, as evident by the large left shoulder of the NMC (104) peak, which is indexed as the RS (200) peak. Once the first temperature hold at 720 °C is reached, the (200) peak starts to decrease as the RS structure is converted back into the NMC phase.

We do not see a (220) reflection for a spinel *Fd*3̅*m* phase, which is consistent with results from Ni-rich NMC structures.^10^ Besides the NMC and RS phases which were present in the STEM images in Figure 2, other phases present in the diffraction patterns include graphitic carbon (C), which is assumed to be trace active graphitic carbon from incomplete separation of the conductive carbon additive in the cathode from the NMC, lithium hydroxide and hydrated lithium hydroxide (LiOH and LiOH·H_2_O), and lithium carbonate (Li_2_CO_3_). The EoL powder has additional peaks that are not indexed (around 2θ = 6 and 7.8), suggesting a different surface phase present than in the relithiated powders. The relithiated sample has LiOH, LiOH·H_2_O, and Li_2_CO_3_ at room temperature, even though no extra Li source was added to this sample, suggesting that the relithiation process leaves trace Li salts on the surface of the particles, as confirmed by NMR and ICP results (Table 1 and Figure S5).

**Table 1:** ICP–OES Results for Cathode Material During Recycling (Adapted from ref 9 ^a^

sample	Li (mol ratio)	Ni (mol ratio)	Mn (mol ratio)	Co (mol ratio)
EoL	0.767	0.649	0.200	0.151
relithiated EoL	1.094	0.650	0.201	0.149
relithated EoL + LiOH, annealed	1.090	0.657	0.195	0.148

a Available under a CC-BY-NC license. Copyright Kirwa et al.).^9^

Also present is a tungsten oxide phase that is stable at high temperatures (WO_3_).^30^ Note that this WO_3_ phase is likely from the furnace setup, which has a tungsten filament that oxidizes and deposits oxide on the outside of the sample vials at high enough temperatures; see Figure S1 for further discussion. It is worth noting that many cathode manufacturers add a conductive coating layer to improve cycling and rate performance; one such coating is a thin, amorphous layer of WO_3_.^31,32^ As the diffraction results suggest, these conductive coating layers may recrystallize during high temperature annealing and affect electrochemical performance, which is an important consideration when picking annealing conditions.

Inductively coupled plasma optical emission spectroscopy (ICP–OES) results, shown in Table 1, were used to make assumptions about atom site occupancies for Rietveld refinement.^9^ A site occupancy of 1 means that site is fully filled with atoms; for sites containing multiple atoms we constrained the sum of the occupancies to be 1. The EoL material starts out with a lithium deficiency, which is replenished during the relithiation process. A small amount of additional lithium is left on the surface after relithiation, as confirmed by NMR results shown in Figure S5 and Table S2, which is why the ratio of Li after relithiation (1.094) is slightly higher than what is found for a pristine NMC 622 material (1.055) in prior work.^9^ We can also see that the commercially cycled NMC 622 composition is closer to a NMC 65–20–15, though for simplicity we will continue to refer to it as NMC 622. In NMC materials, there is a correlation between higher capacity and higher nickel content, which is consistent with our findings in Figure 1b where the fully annealed NMC 65–20–15 material (with LiOH) recovers a slightly higher capacity compared to the pristine 622 material.^33^

The ICP mole ratios of transition metals Ni, Mn, and Co do not vary significantly during the recycling process. Therefore, Mn and Co occupancies were fixed at 0.20 and 0.15, respectively, for the NMC phase. Total Ni occupancies (Ni_Li_ + Ni_TM_) were set to 0.65, and total Li occupancies (Li_Li_ + Li_TM_) for EoL material and relithiated material was 0.77 and 1.00, respectively, for the NMC phase. Of course, lithium can leave vacancies during annealing, as evidenced by the formation of Li_2_CO_3_ and reported in the literature.^10,12^ However, synchrotron data has limited signal from lithium, so we cannot distinguish between lithium occupancies and site vacancies in the lattice. Therefore, lithium occupancies are more qualitative. It was assumed that, when cation mixing occurred, one lithium would switch places with one nickel atom, so Ni_Li_ = Li_TM_. For the RS phase, the ratios of Ni/Mn/Co were fixed to 0.65/0.20/0.15, and the transition metal site allowed for lithium occupancy, using 1-Li_TM_ = Ni_TM_ + Mn_TM_ + Co_TM_. The oxygen occupancy for the trigonal NMC and cubic rock-salt phase was fixed at 1, because refinement of oxygen occupancies is not as accurate for synchrotron data.^34^

Figure 4 presents a summary of the Rietveld refinement results. Figure 4a shows the phase composition of all three powders during annealing. Both relithiated samples start out with higher RS content than the EoL sample, indicating that the redox relithiation process creates some of the RS phase, which explains why it is crucial to anneal after relithiation to correct the crystal structure. Previous literature has shown that the washing step, which is vital to perform before relithiation, can dissolve the transition metals and cause surface RS phase to form.^35^ From Figure 3c, the RS (200) peak increasing in intensity with temperature correlates with a decrease in NMC wt % in Figure 4a at 500–720 °C. This conversion between NMC and RS phases is typical for these materials at high temperatures and indicates that the RS structure is an important intermediate step in the formation process.^10,36–38^ The relithiated + LiOH sample appears to undergo this phase transition at lower temperatures than the other samples. Both relithiated samples have lower amounts of RS after annealing than they started with. The relithiated + LiOH sample after annealing has 3.6 wt % RS, which is similar to pristine materials.^12^

**Figure 4:**
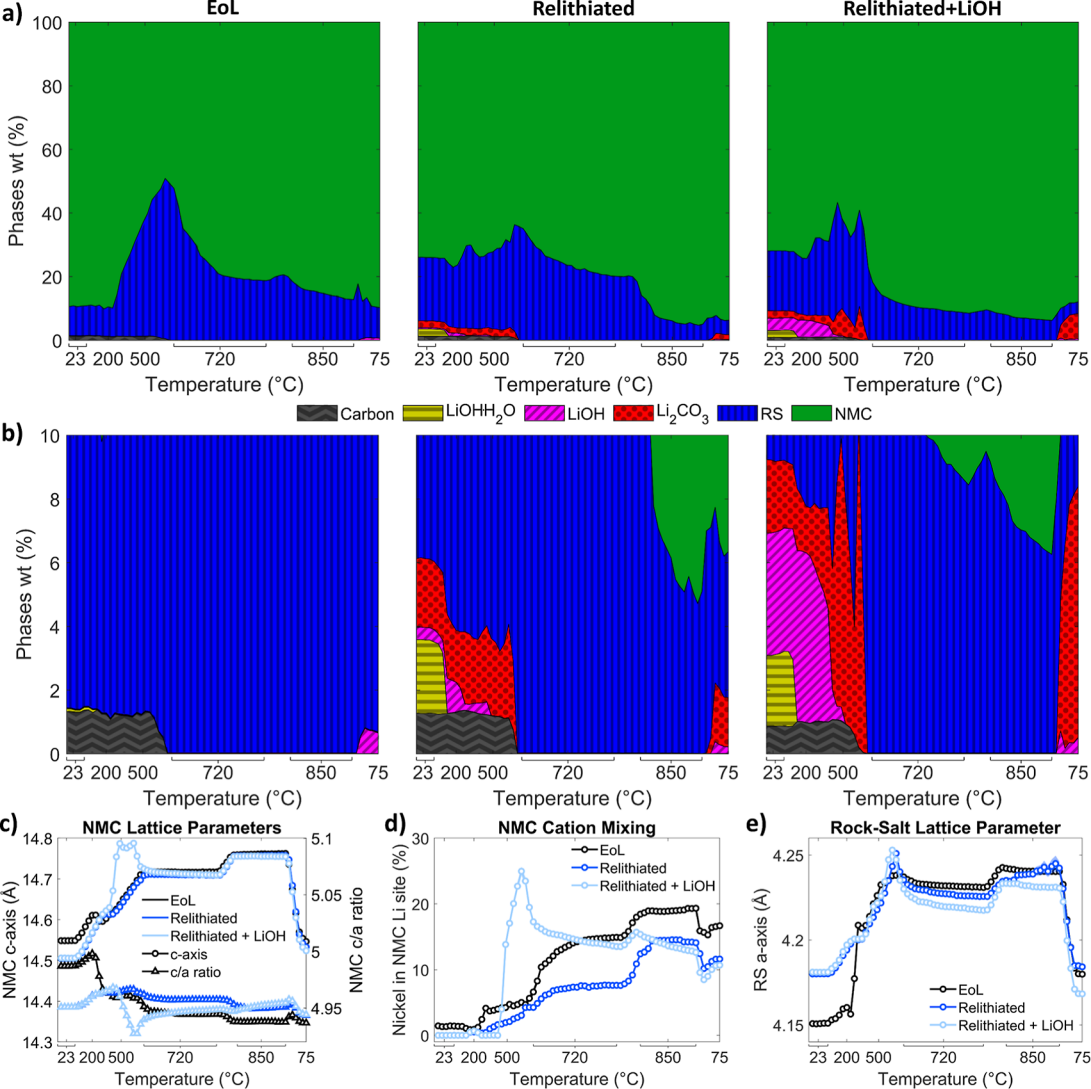
Rietveld result summary. (a) Phase composition of each powder during annealing. (b) Zoomed-in phase composition plots, showing impurity phase amounts more clearly. (c) Lattice parameters *a* and *c* for the NMC phase during annealing, and the *c*/*a* ratios for the NMC phase for each sample during annealing. (d) Cation mixing (Ni occupancy in Li site) for the NMC phase. (e) Rock-salt lattice parameter. For c–e, shaded areas indicate confidence intervals for the parameters.

A zoomed-in portion (total wt % < 10%) is shown in Figure 4b for better clarity. Both relithiated samples start with small amounts of Li_2_CO_3_ and LiOH·H_2_O, which are left over on the surface from the relithiation process. The LiOH·H_2_O phase converts into LiOH above 100 °C as the water evaporates. As seen in Figure 4b, the LiOH phase disappears at around 450 °C, which is consistent with the melting temperature of LiOH. After this temperature, Li_2_CO_3_ forms, likely due to the CO_2_ in the air reacting with the lithium from melted LiOH. Then, when the temperature hold of 720 °C is reached, the Li_2_CO_3_ peaks disappear (which correlates with the melting temperature of Li_2_CO_3_). Once the relithiated samples are cooled down, Li_2_CO_3_ precipitates out. The relithiated + LiOH sample starts out with an additional 3.5 wt % of LiOH, which is a bit higher than the 1.8 wt % LiOH added to this sample, which might be due to the small sample size of the in situ annealing setup. The WO_3_ impurity phase, which comes from the furnace filament and recrystallizes at temperatures above 800 °C, is not included in the phase composition plots in Figure 4.

The melting points of LiOH and Li_2_CO_3_ correlate with decreases in the RS phase in both relithiated materials. The RS phase has been shown to have slower lithiation kinetics, which causes lithium evaporation and is why additional lithium sources like LiOH and Li_2_CO_3_ are crucial for successful annealing.^12,38^ The phase transition of the relithiated + LiOH RS phase likely happens at lower temperatures compared to the relithiated RS phase due to the excess of LiOH added. This suggests that the trace lithium salts left behind on the relithiated (without added LiOH) sample are not sufficient to drive this phase transition, which is why adding additional lithium salts with a low melting temperature is required. Interestingly, the RS phase amount of the EoL material also decreases after sufficient heating, despite not having any excess of lithium in this sample. Studies have shown the importance of an oxygen-rich environment for lithiation of the RS phase, in order to convert it back to the layered oxide phase; since these samples were annealed in air there was a source of oxygen for this phase transition.^38,39^ However, this mechanism still requires a lithium source; while it is possible there are lithium sources in the EoL powder that are not crystalline (and therefore do not show up in diffraction patterns), this does not seem to fully explain the significant decrease in RS phase amount in the EoL sample. It is possible that the lithium from the layered oxide phase moves to relithiate the RS phase, which could explain the high cation mixing values shown in Figure 4d, which correlate both to lithium switching with a nickel atom as well as lithium vacancies (as it is hard to refine lithium vacancies for synchrotron data).

The *c*-axis lattice parameter and the *c*/*a* ratios of the NMC phase are shown in Figure 4c (the *a*-axis lattice parameter is shown in Figure S6). In the NMC layered oxide structure, the *c*/*a* ratio increases as the quality of the oxide layering increases.^40^ This ratio is lower for the relithiated powders before annealing than the EoL powder, which indicates the relithiation process damages the layering of the recycled material if the structure is not annealed. The relithiated powder *c*/*a* ratio stays constant throughout annealing. Once the EoL and relithiated + LiOH samples are heated to above 250 and 450 °C, respectively, there is a sharp decrease in the *c*/*a* ratio, implying at these temperatures the layered oxide structure is destabilized. The *c*/*a* ratio of the EoL material continues to decrease as the temperature increases, and the EoL material ends up with a worse layering quality than both relithiated materials after annealing. Therefore, annealing without relithiating the powder has destroyed the layered oxide structure.

The cation mixing amount (defined as the Ni occupancy in the NMC lithium site) is shown in Figure 4d. EoL material starts out with higher cation mixing levels than the relithiated material, indicating the relithiation process has replenished the lithium, which limits cation mixing. For all samples, the NMC *c*-axis increases with temperature as expected due to thermal expansion. Cation mixing for all samples increases as temperature increases up to the first temperature hold, indicating ion movement and nickel ion reduction due to heat treatment. The cation mixing amounts for all samples after annealing are higher than what is typical in pristine materials (>5%).^12^ The relithiated + LiOH sample ends up with the lowest mixing amount. The usual annealing time is 8 h to get comparable electrochemical performance to pristine material (as shown in Figure 1). However, since beamtime was limited, the in situ synchrotron diffraction experiment only had ∼1 h annealing times.

At ∼500 °C, the relithiated + LiOH material has a much higher *c*-axis and higher cation mixing amount than the other materials at this temperature. This spike in lattice parameter and cation mixing happens soon after LiOH melts at around 450 °C. An expanded *c*-axis and higher cation mixing indicates a more disordered structure. This disorder decreases significantly once the lower temperature hold of 720 °C is reached. The presence of this more disordered NMC in the relithiated + LiOH sample indicates that a more disordered phase is required for faster restructuring and is only energetically favorable once the Li source has melted to provide additional lithium to make up for lithium evaporation.

Once the temperature reaches 720 °C, the relithiated + LiOH *c*-axis NMC disorder decreases significantly, and the *c*-axis and cation mixing levels return to comparable values to the other materials. During the 720 and 850 °C holds, the *c*/*a* ratio of the relithiated + LiOH material is increasing, and the cation mixing amount is decreasing, signaling that the crystal structure quality of the NMC phase is improving during the annealing holds. The other two samples have *c*/*a* ratios and cation mixing levels that are stable or deteriorating during annealing, further supporting the need for the extra Li source during annealing. As the relithiated + LiOH material crystal structure is improving during the annealing holds, this suggests it could return to pristine levels if the annealing step was held for long enough times. If we linearly extrapolate the decreasing slope of the cation mixing for the relithiated + LiOH sample during both temperature holds, we can estimate that the cation mixing of the relithiated + LiOH sample would reach 5% after 5.5 h at 720 °C and after 3.4 h at 850 °C. This correlates well with the results from Figure 1, which show than annealing times longer than 4 h at 720 °C are needed for electrochemical performance comparable to pristine.

In lithiated nickel-rich RS phases, a higher *a*-axis corresponds to a lower lithium content in the phase.^41–43^ Figure 4e plots the RS lattice parameter during annealing (additional RS phase parameters are shown in Figure S6). Most of the increase in lattice parameter is due to thermal expansion (like the NMC structure). A small spike in the RS lattice parameter happens above 500 °C for both relithiated materials; therefore, at this temperature, the RS phase has less lithium content. The EoL material has no added lithium from salts or relithiation, which results in a lithium deficient RS phase, as seen by its higher *a*-axis compared to the relithiated RS phases. Interestingly, the RS phase lattice for the relithiated + LiOH material is decreasing during the annealing temperature holds and ends up at a lower value than the other two samples after cooling down. This suggests that the RS phase is being lithiated during these holds, which is why an additional Li source is important because some of the lithium inventory is lost to lithiating the RS phase.

Importantly, there does not appear to be a significant difference between the two temperature holds, because the relithiated + LiOH structure seems to recover its layered oxide structure at 720 °C. There might be differences in kinetics between the 720 and 850 °C holds, as evidenced by the difference in slope in the NMC cation mixing for the relithiated + LiOH material during both holds. However, both the lattice and the cation mixing levels are trending in the correct direction, so a slightly longer annealing time at the lower temperature hold should be sufficient to anneal the structure. It is also evident that the EoL material has more cation mixing and worse oxide layer quality after high temperatures than relithiated material. This indicates that if the prior relithiation step is insufficient, the structure will be more damaged by the annealing step instead of properly converted back to pristine.

The annealing step could affect more than just the NMC crystal structure, however. The NMC microstructure (primary and secondary particle sizes, particle internal porosities) could also be affected by high temperatures, which is important as many cathode manufacturers carefully control these parameters to control electrochemical performance.^10,15,16^ STEM cross-sectional images of cathodes at various stages of recycling are shown in Figure 5a. Note that the pristine NMC 622 sample is from a different source than the actual pristine material that corresponds to the EoL (and recycled) samples, so the variations in particle morphology might not be due to battery cycling but differences in starting material. Both the EoL and relithiated electrodes have a porous electrode morphology while the annealed electrode is denser, with primary particles more closely packed.

**Figure 5:**
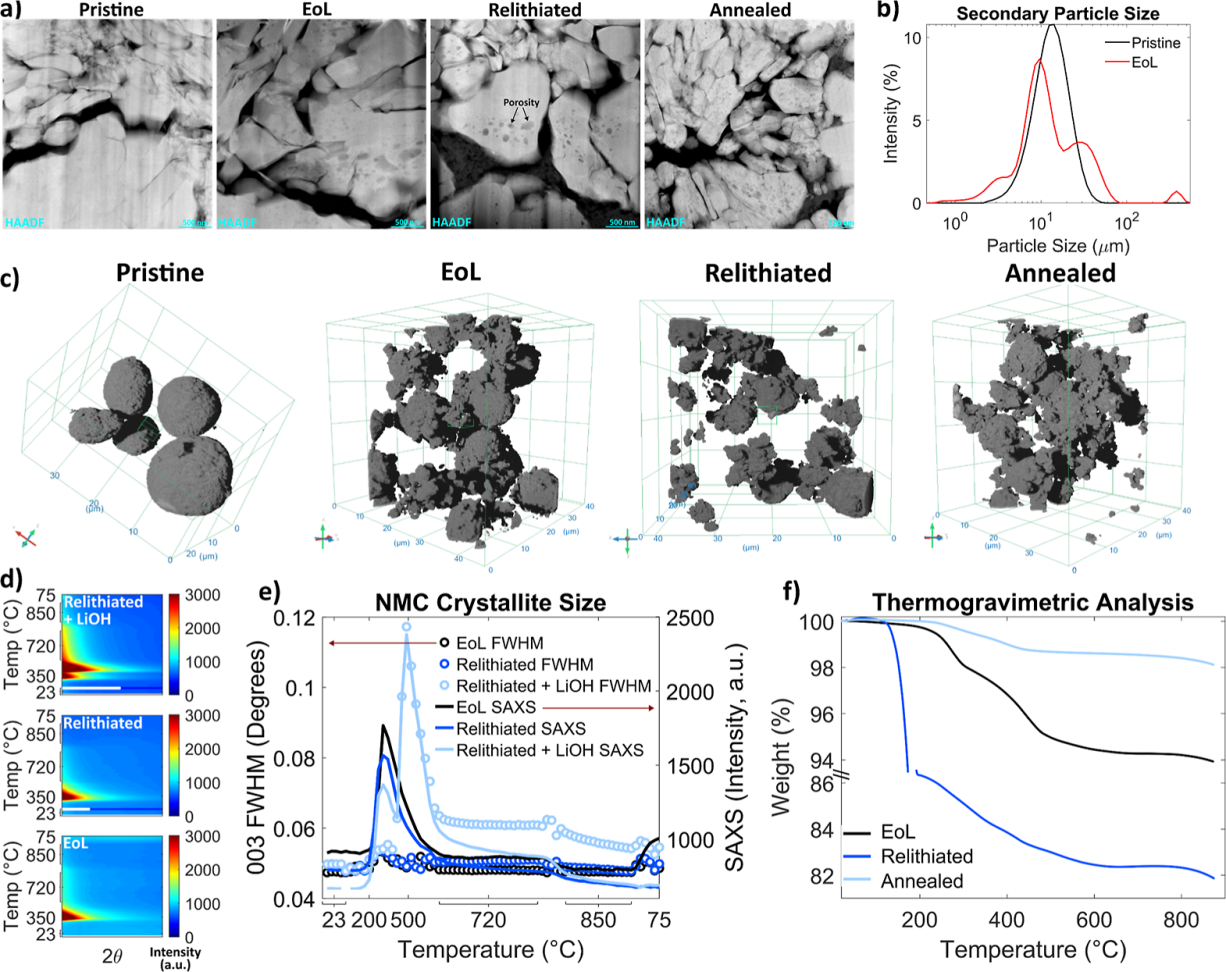
NMC microstructure. (a) HAADF STEM electrode cross-sectional images of the pristine, EoL, relithiated, and annealed material. (b) Secondary particle sizes for pristine and EoL material. (c) 3D tomography images of powders. (d) Diffraction patterns of the small-angle scattering (2θ < 1.5) for each sample. (e) Integrated peak area of the (003) NMC peak, and small-angle scattering peak area. (f) TGA results for EoL, relithiated, and annealed samples.

Cathode manufacturers typically control secondary particle structure and size by structuring primary crystallites into larger, spherical agglomerations, which allows for better electrode morphologies without compromising electrochemical performance.^15,16^ The particle size distribution, measured with a particle size analyzer, of pristine and EoL material is shown in Figure 5b. The EoL material has larger particles that are likely clumps of multiple particles, as well as smaller particles, that are probably fractured secondary particles. Full-field transmission X-ray microscopy (TXM) was performed on the pristine, EoL, relithiated, and annealed material. Figure 5c shows the reconstructed 3D volumes for each sample. The particle size differences between pristine and EoL material is obvious, but it does not appear that the secondary particle sizes change significantly throughout the recycling process.

The primary particle size is also significant, because ion diffusion can be limited in larger primary particles.^16^ Crystallites can also regrow during high-temperature steps, which changes the size of the primary crystallites and could affect electrochemical performance. To estimate the primary particle size of the layered oxide structure, we can use small-angle scattering (SAXS), where the presence of small nanoparticles increases SAXS intensity.^38,44,45^ The small-angle scattering for each sample during annealing is shown in Figure 5d. Plotted in Figure 5e is the total small-angle scattering area, which is the integrated area of the intensities 2θ < 1.5. Also plotted in Figure 5e is the full-width-half-max (fwhm) of the NMC (003) peak throughout annealing, as it is hard to refine crystallite size using Rietveld analysis. Peak broadening can be attributed to both changes in crystallite size and lattice strain. The corresponding peaks in small-angle scattering and (003) fwhm for the relithiated + LiOH sample from 450 to 720 °C indicate the relithiated + LiOH NMC phase converts into nanosized crystallites at these temperatures. This corresponds to the more disordered structure (high cation mixing, large *c*-axis) at these temperatures shown in Figure 4. These disordered, nanosized primary particles might be easier to lithiate and could be the reason why the relithiated + LiOH sample experiences improvements in cation mixing and lattice parameters. The relithiated + LiOH material has a larger NMC (003) fwhm after annealing than before annealing, suggesting that the primary particle size decreases slightly after annealing, which might be due to the nanosized crystallites that formed at 450–720 °C. This could also be due to incorporation of lithium and oxygen during the relithiation and annealing process.^10,16^ This change in primary particle size could pose challenges for chemical direct recycling methods if this parameter needs to be preserved throughout recycling, but electrochemical results indicate recycled material has similar rate performances to pristine, so this small change might not be an issue.^9^

As illustrated in Figure 5a, particle internal porosity increases after annealing, possibly due to thermal decomposition of the NMC material. Oxygen vacancies, which are found in degraded NMC, can accelerate thermal decomposition of the NMC structure, which means if EoL material is not properly relithiated before the annealing step, the NMC structure could be unstable.^13,46^ All samples have an increase in small-angle scattering from 250 to 550 °C, which could be related to the growth of the nanosized RS phase (Figure S6). Also, previous studies showed that NMC experiences exothermic reactions 240–290 °C, that could be attributed to O_2_ release from NMC decomposition.^47–49^ Around 400–500 °C, the typical cathode binder polyvinylidene fluoride (PVDF) decomposes, which could be present in trace amounts in these samples.^9,47^ These decompositions correlate with the increased small-angle scattering at these temperatures. A more in-depth investigation into the relationship between SAXS and thermal decomposition is left for future research.

Thermogravimetric analysis (TGA) results for EoL, relithiated, and annealed samples are shown in Figure 5f. The large mass loss at 180 °C for the relithiated sample is 2,5-di-*tert*-butyl-hydroquinone (DTBHQ) decomposition, as confirmed by Fourier transform infrared spectroscopy (FTIR) results (see Figure S7). This is likely leftover from the redox relithiation process. Mass losses from 250 to 300 °C for all samples match the thermal decomposition temperatures related to O_2_ release from NMC. Additional mass losses at around 400 °C match PVDF decomposition. The annealed sample loses the least amount of mass during heating, indicating it is the most stable structure, which correlates with its better electrochemical performance.

X-ray absorption near edge structure (XANES) was performed simultaneously with the TXM imaging. This technique uses the tunability of synchrotron incident X-ray energies to resolve 3D tomography volumes while mapping elemental composition. The absorption coefficients of different elements experience a step change at their characteristic absorption edges (such as the K-edge).^22,50^ Therefore, elemental maps can be obtained from the difference between absorption intensity at incident energies above and below the K-edge for each element. Ni XANES maps are then fitted linearly using reference materials of LiNiO_2_ (for Ni^3+^) and NiO (for Ni^2+^) to resolve relative oxidation states. The K-edge for Ni absorption edge shifts to higher energy values for a higher oxidation value; in other words, Ni^3+^ has a higher maximum absorption intensity of the K-edge than Ni^2+^.^51^ Figure 6a shows the Ni oxidation state map for each sample, where a brighter yellow color indicates more relative Ni^3+^ character at that location. Particles have internal porosity, as seen from the STEM images in Figure 5a. There is a slight variation in Ni oxidation state with the depth of the particle, where the outside of the particle tends to have a higher oxidation state than the inside. Figure 6b shows the elemental maps for each sample; atoms appear well mixed.

**Figure 6:**
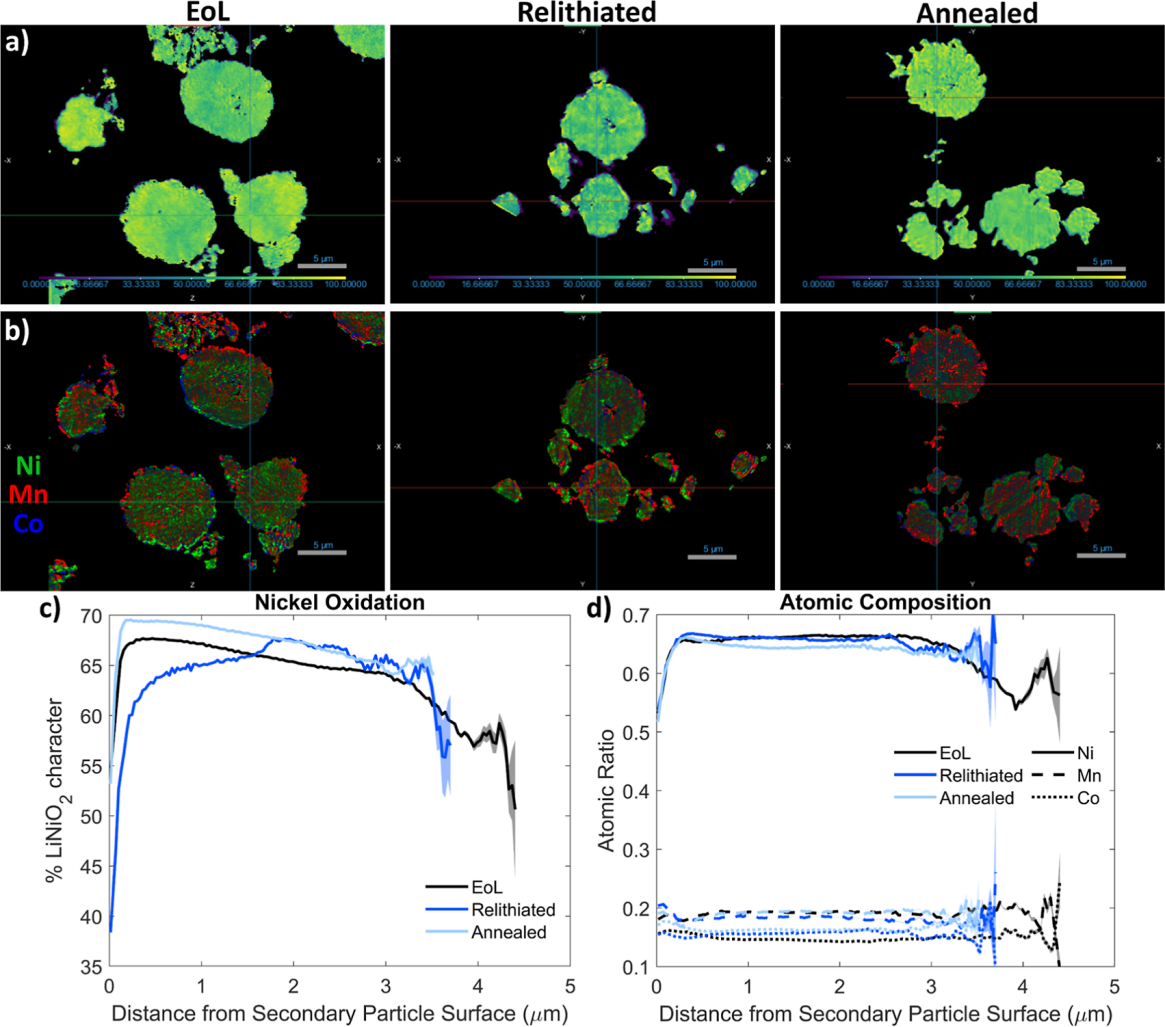
TXM and XANES results. (a) Maps of Ni oxidation state, where a higher value (shown as a brighter yellow) means more Ni^3+^. Particles are cropped to reveal internal structure. (b) Elemental maps for each sample. (c) Depth-dependent Ni oxidation state results, with shaded regions indicating a 99% confidence interval. (d) Depth-dependent atomic ratios for each sample, with shaded regions showing a 99% confidence interval.

Depth-dependent statistics of the Ni oxidation state and atomic ratios were calculated for all samples, following a similar procedure reported previously by Allen et al.^22^ These statistics are calculated by isolating five secondary particles of similar size, calculating their surfaces, and measuring the mean values as a function of depth from particle surface to core through Euclidean distance mapping with 99% confidence intervals. Particle surface is mapped through a surface mesh of the original segmentation. Figure 6c plots the percentage of LiNiO_2_ character (from the K-edge peak locations compared to the reference LiNiO_2_ and NiO samples) versus depth from the secondary particle surface, averaged across the five particles. More LiNiO_2_ character indicates a higher oxidation state approaching Ni^3+^, whereas more NiO character indicates a lower oxidation state approaching Ni^2+^. Figure 6a illustrates there is a slight core–shell structure with Ni oxidation state, where more Ni^3+^ is on the outside of the particle, which matches the higher LiNiO_2_ character at the surface of particles for the EoL and annealed samples. The relithiated sample has more Ni^2+^ content on the surface of the secondary particle than the EoL sample, suggesting that chemical relithiation has primarily affected the surface. The annealed sample has the highest Ni^3+^ content, which explains its superior electrochemical performance, and correlates with the results shown in Figure 4d, which indicate annealing fixes the cation mixing present in the structure after chemical relithiation. However, while Figure 4 shows results from bulk powder diffraction measurements, the XANES results demonstrate that there is a depth-dependency to the Ni oxidation state that needs to be taken into account. In particular, the material after chemical relithiation has lower oxidation state on the surface of the particle. While this could indicate structural disorder that has been shown to reduce capacity and cyclability due to higher surface impedance, the bulk disorder we have quantified in our Rietveld refinement is only slightly higher than the average bulk pristine NMC 622 reported in literature, and no detrimental effects in electrochemical performance were observed.

Figure 6d plots the Ni, Mn, and Co ratios as a function of depth. There is some variation at the surface of the particle, where this method is less accurate in mapping elemental composition.^22^ Atomic ratios do not vary significantly throughout the depth of the particle, nor do the ratios change significantly throughout the recycling process, which is also confirmed by the ICP data shown in Table 1. See Figures S8 and Figure S9 for additional images and results from the TXM and XANES experiment.

Therefore, as atomic ratios are uniform throughout the depth of the particles across all samples, differences in Ni oxidation state can be confidently assigned to Li and/or O loss. This corresponds well with the synchrotron diffraction results, which show that annealing with LiOH corrects the NMC layered oxide structure. Taken together, the results in this paper suggest that a chemical relithiation step, followed by a suitable annealing step, is sufficient to repair the capacity degradation found in end-of-life material. However, other cathode parameters like internal porosity and crystallite size might be affected by this annealing step, which is an important consideration for this direct recycling method if the source material is changed too much from its pristine condition during annealing. If, for example, a source material has been designed to have no internal porosity, annealing might introduce some porosity that might affect electrode processing or electrochemical performance. Nonetheless, the material studied in this paper does not seem to be negatively affected by slight changes in particle structure due to annealing, and an adequate relithiation step prior to annealed should stabilize the structure enough to limit changes.

## Conclusions

In summary, we characterized the crystal structure of end-of-life and relithiated NMC 622 cathode material during high-temperature annealing using multiple characterization techniques, including synchrotron powder diffraction and Ni XANES 3D mapping. Degraded NMC material is not stable at high temperatures without a relithiation procedure. During annealing, it is necessary to add an additional source of lithium to overcome lithium evaporation and form a more disordered, nanocrystallite, intermediate structure that is easier to relithiate and recrystallize at high temperatures. As the critical structural transition between the disordered intermediate structure into the desired layered oxide structure happens at above 550 °C, an annealing temperature of 720 °C is more than sufficient to fully convert the cathode crystal structure after chemical relithiation back to pristine. We also show that the primary particle size and internal particle porosity can be affected by the annealing step, and bulk transition metal ratios and secondary particle size seem unaffected. This is a significant finding to help the battery recycling community to understand how the high-temperature annealing step that is crucial to chemical relithiation strategies affects the particle structure and how successful this direct recycling strategy can be for different source materials.

## Supplementary Material



## Notes

Extended STEM results, diffraction patterns, residuals from Rietveld fits, monoclinic phase discussion, NMR results, additional Rietveld parameters, FTIR results, extended Ni-XANES results (PDF)

## #

K.Z.P. Deceased on May 9, 2024.
