# Supplementary material for: Effect of Annealing on Direct Recycled NMC Cathodes
Source: Chem Mater. 2025 Nov 24;37(23):9406–19. doi: 10.1021/acs.chemmater.5c01824 (PMC12874368; doi:10.1021/acs.chemmater.5c01824)
Supplement: Supplementary file 1 [file cm5c01824_si_001.pdf]

The Effect of Annealing on Direct Recycled NMC Cathodes

Juliane I. Preimesberger,[1] *Cyrus K. Kirwa,*[1,2] *Eva Allen,*[3] *Nikita S. Dutta,*[1] *Evelyna Wang,*[3] *Fulya Dogan,*[3] *Patrick Walker,*[1] *Yaocai Bai,*[4] *Krzysztof Z. Pupek,*[5♦] *Lisa Stanley,*[1] *Tiffany L. Kinnibrugh,*[3] *Matthew Nisbet,*[3] *Timothy T. Fister,*[3] *Hongmei Luo,*[2] *Jaclyn E. Coyle*[1*]

1. Materials, Chemical, and Computational Science Center, National Renewable Energy Laboratory, Golden, Colorado 80401, USA
2. Department of Chemical and Materials Engineering, New Mexico State University, Las Cruces, New Mexico 88003, USA
3. Chemical Sciences and Engineering Division, Argonne National Laboratory, Lemont, Illinois 60439, USA
4. Electrification and Energy Infrastructures Division, Oak Ridge National Laboratory, Oak Ridge, Tennessee 37830, USA
5. Applied Materials Division, Argonne National Laboratory, Lemont, Illinois 60439, USA

*Corresponding Author. Email Jaclyn.Coyle@nrel.gov
♦K.Z.P. Deceased on May 9, 2024

Additional STEM results are shown in Figure S1 for pristine NMC 622 (Targray), the EoL material, the relithiated material, and the annealed material. Figure S1a shows the disordered surface layer for all samples, Figure S1b shows the rock-salt structure, and Figure S1c shows evidence of cation mixing. All samples have a layered structure consistent with the NMC phase. The pristine material has smaller crystal grains compared to other samples (which is likely more due to differences in source material instead of effects of degradation or recycling). All samples have a disordered surface, with the rock-salt structure present. Cation mixing is the most obvious in the relithiated sample, but because STEM images such a small area, the synchrotron diffraction results are likely more accurate in measuring cation mixing quantitatively.

Elemental analysis using STEM of the EoL material (as shown in Figure S1d) also confirmed that the EoL material does not start out with a layer of WO_3. While there is W in the sample, it is present in a similar amount to, and co-located with, the Pt, both of which are contaminants from the FIB sample preparation. The W does not show up in an even coating layer, indicating that the WO_3 phase found in the Rietveld analysis in Figure 4 is most likely from the experimental setup of the furnace with a tungsten filament, which is known to deposit WO_3 onto the capillary tubes during heating.

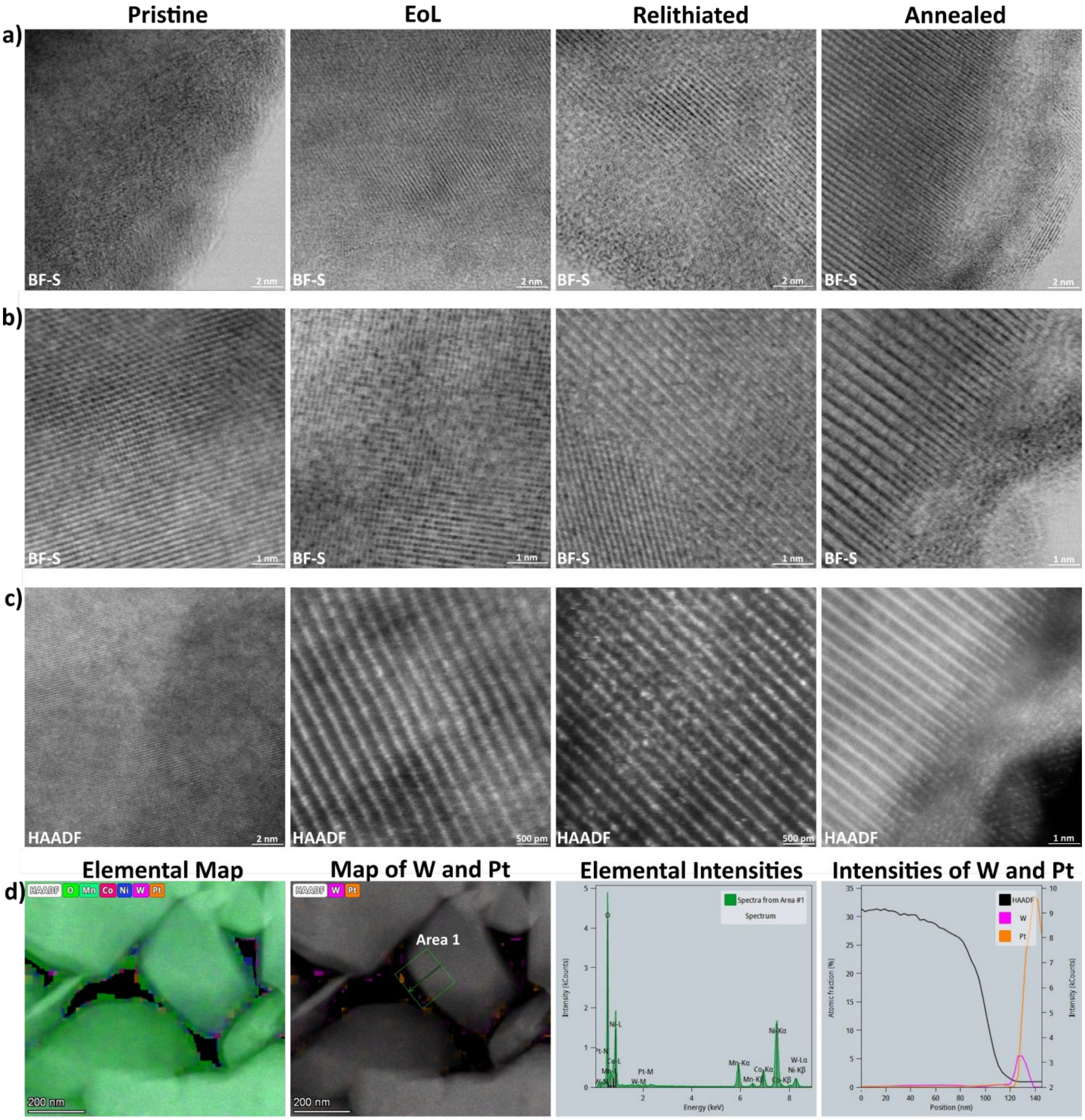

Figure S1: STEM results for all samples. a) The disordered surface layer for all samples. b) Evidence of a rock-salt structure. c) Cation mixing (bright spots in the dark lithium layer for a HAADF image). d) Elemental analysis of the EoL material.

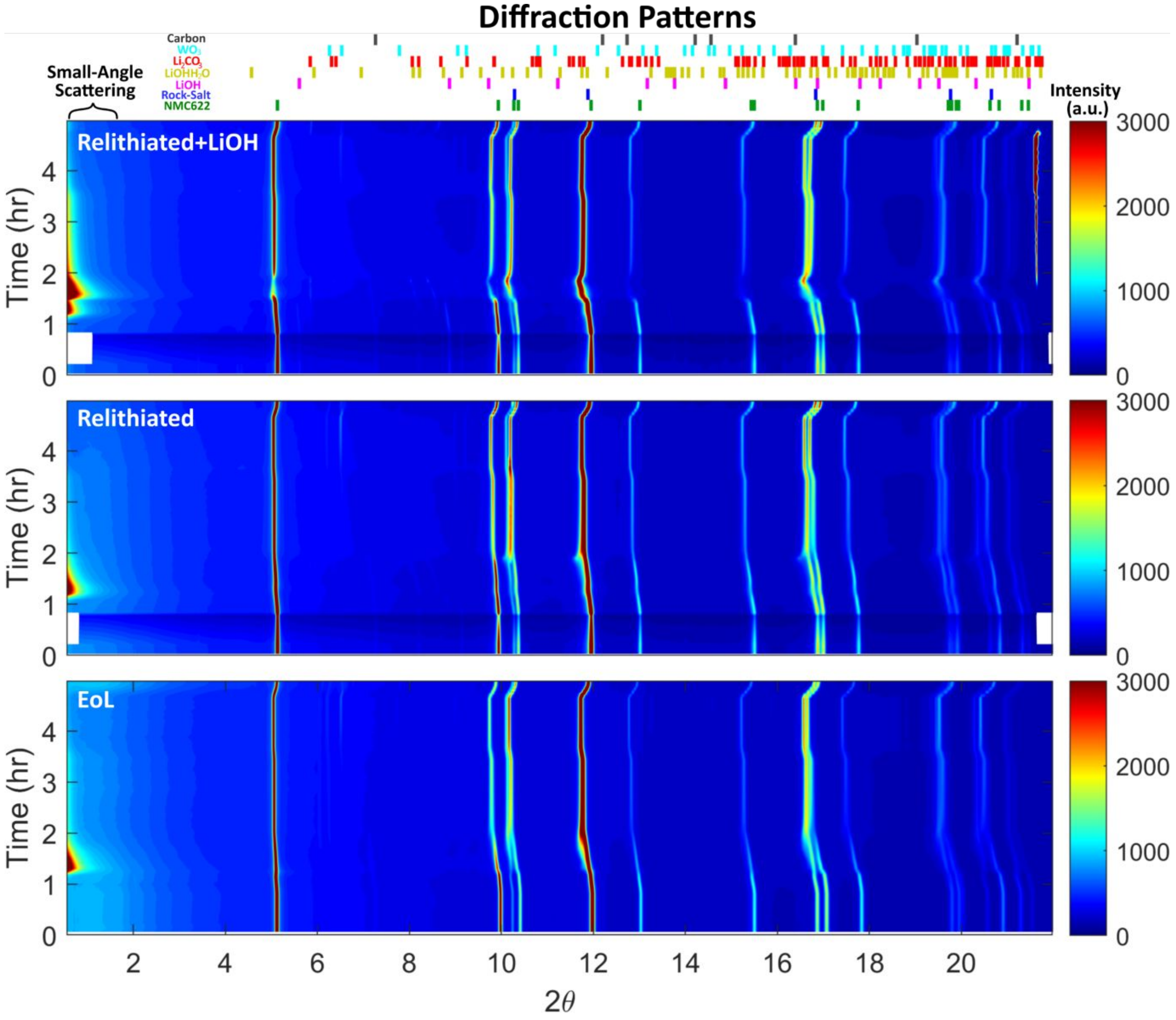

Figure S2: Full diffraction patterns, with peak locations (from the room-temperature Rietveld fit for the relithiated samples) marked.

The full diffraction patterns of all three synchrotron samples are shown in Figure S2. Peak locations (taken from the Rietveld refinement of the relithiated material at room temperature before annealing) are marked for each phase. The residuals for all Rietveld fits are shown in Figure S3.

There might be an intermediate phase that has peaks that overlap with the NMC and RS phases and has been noted in other XRD studies looking at NMC EoL material [1]. It is possible this is a monoclinic structure, which is known to appear in lithium cobalt oxide (LCO) and NMC cathode materials due to local lithium vacancies [2,3,4,5]. An attempt to fit a monoclinic structure, matching previous reports, to the EoL diffraction patterns was made [3]. This monoclinic structure does not include cobalt, but since synchrotron diffraction cannot easily distinguish between cobalt, manganese, and nickel, this is unlikely to affect the fit quality but might help explain why the lattice parameters refined to different values than what was previously published.

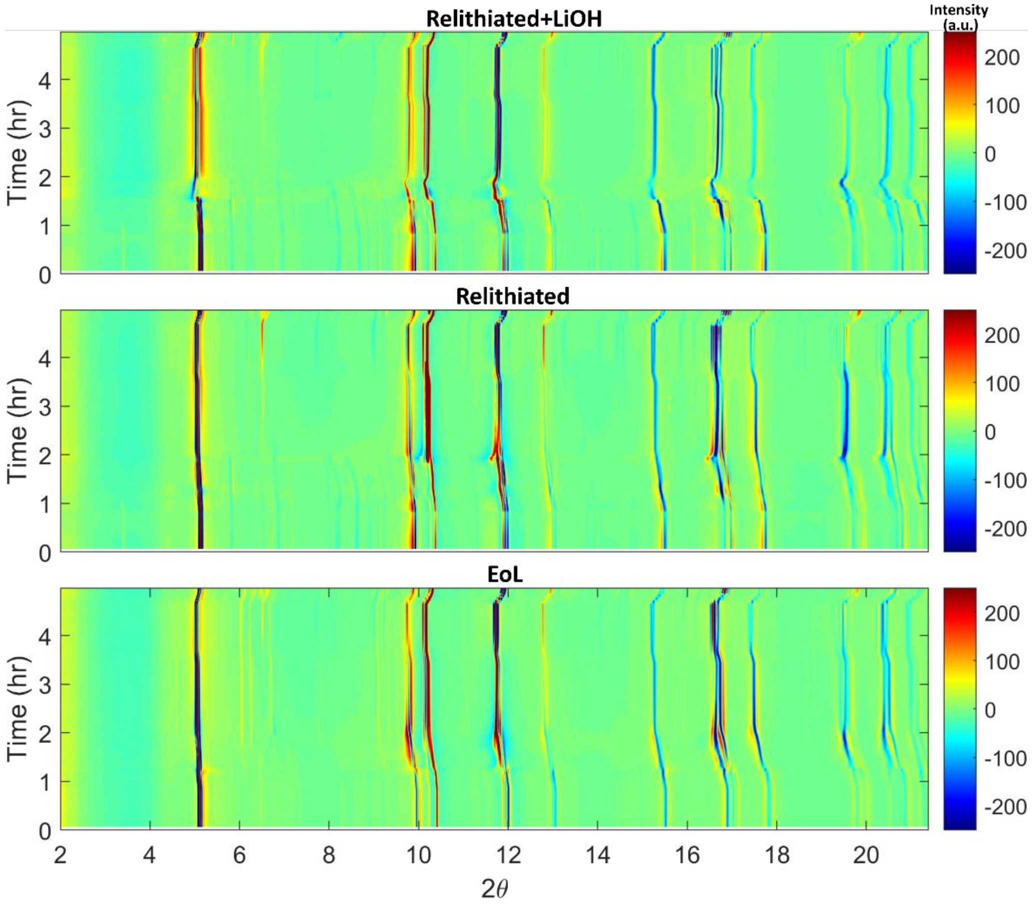

Figure S3: Residuals from Rietveld fit.

Figure S4 presents the Rietveld refinement results for the EoL sample during annealing, including this monoclinic phase. Figure S4a shows that the χ^2 values are slightly improved when including the additional phase, particularly in the temperature range 200-720 ℃. Figure S4b shows the phase amounts including the monoclinic phase. A significant amount of this monoclinic phase is present between 200-720 ℃, potentially indicating that there could be a phase transition at these temperatures. Figure S4c is a plot of the monoclinic phase cell volume during annealing, which mostly changes due to thermal expansion. Figure S4d and Figure S4e plot the NMC phase lattice and cation mixing parameters, from refinements with and without the monoclinic structure. Neither the lattice nor the cation mixing parameters change significantly for the NMC phase, indicating that our conclusions based on Rietveld fittings without the monoclinic phase are still valid.

Another possible explanation of the conversion of the EoL RS phase back into a layered oxide phase without added lithium is this monoclinic phase. As seen in Figure S4b, including this phase in the refinement reveals that the RS phase, once formed, does not substantially disappear. Instead, the monoclinic phase is converted back into the layered oxide phase. It has been shown in $LiNiO_2$ systems that the monoclinic phase is capable of transforming back into a

hexagonal phase with additional lithium loss [6]. However, if the layered oxide phase in Figure S4b includes both lithiated and delithiated hexagonal phases, the amount of lithium loss (or cation mixing) should be pretty significant.

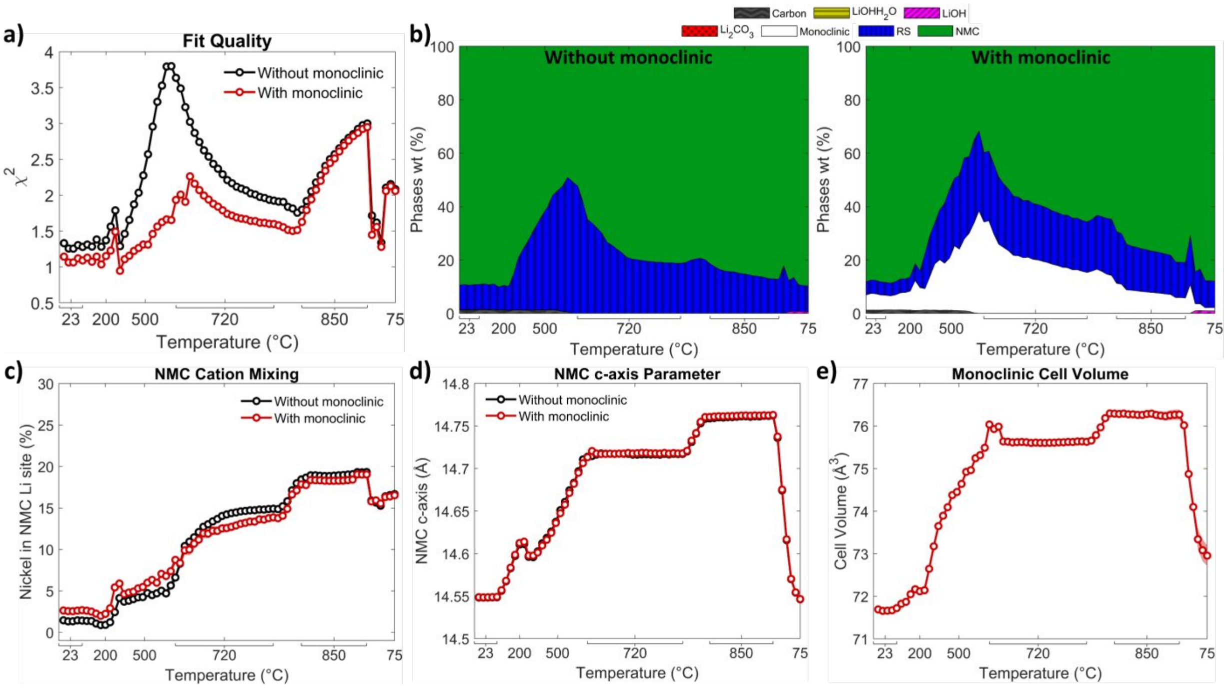

Figure S4: The effect of including a monoclinic phase in the Rietveld refinement for the EoL sample during annealing. a) χ^2 values, with and without the monoclinic phase. b) Phase wt %, with and without the monoclinic phase. c) Monoclinic phase cell volume during annealing. d) NMC c-axis lattice parameter, with and without the monoclinic phase. e) NMC cation mixing amount, with and without the monoclinic phase.

Table S1 reports the monoclinic structure refinement results from the room temperature, unannealed, EoL sample. All cell dimensions are fairly different from previously reported monoclinic structures; in particular, the a-axis, b-axis, and c-axis reported in Table S1 are all larger (a is 5.04440 in this work compared to 4.96477, b is 2.93255 compared to 2.86429, and c is 5.11086 compared to 5.07119 from Arachi et al. [3]). This difference is why the authors have chosen not to include the monoclinic structure in the main paper, because proposing a different monoclinic crystal structure should be left to a more in-depth future study. Additionally, we do not see evidence of a monoclinic phase in the STEM data, though it might primarily occur during heating, which would not be captured in the STEM data. Regardless, the authors acknowledge that an additional structure such as the monoclinic structure reported in Table S1 might explain why the χ^2 values of fits without this phase are a little high during heating.

Table S1: Monoclinic phase parameters and atomic positions (room temperature, EoL sample).

$Li_{0.7}Ni_{0.5}Mn_{0.5}O_2$ (5.7 wt %), $Li_{1-x}NiO_2$ (dis) type structure (modified from [3]**)**

Space group: C 1 2/m 1, $a = 5.04440$, $b = 2.93255$, $c = 5.11086$, $\beta = 108.51$

Atomic Positions

Atom	Site	x	y	z	Occupancy
Li	$2a$	0	0	0	0.2516
Ni	$2a$	0	0	0	Li_{2d}
Li	$2d$	0	0.5	0.5	0.00
Ni	$2d$	0	0.5	0.5	0.50- Li_{2d}
Mn	$2d$	0	0.5	0.5	0.50
Li	$4i$	0.37	0	0.124	0.178
O	$4i$	0.2221	0	0.718	1.00

$^{6,7}Li$ solid-state magic-angle-spinning (MAS) NMR data are shown in Figure S5. Samples measured were EoL, relithiated, annealed, and annealed with 7 mol% LiOH. The EoL sample was washed with 0.5M LiOH in deionized water (before relithiation, all relithiated and annealed samples also had this washing step). Figure S5a shows the ^6Li spectra, which shows a peak shift to the left for the relithiated or annealed samples compared to EoL, indicating successful relithiation. Figure S5b shows the ^7Li spectra, where there is bulk lithium (present in the NMC layered oxide structure) as well as surface lithium. A close-up of the surface lithium peak around 0 ppm is shown in Figure S5c. A peak shift to the left is visible after annealing, like in the ^6Li spectra. Relative amounts of bulk versus surface lithium from the ^7Li spectra (normalized by mass) were quantified by integrated peak areas and reported in Table S2. The relithiation step adds additional surface lithium compared to the EoL sample, which is removed after annealing. Annealing with extra LiOH increases the surface lithium slightly compared to annealing without LiOH.

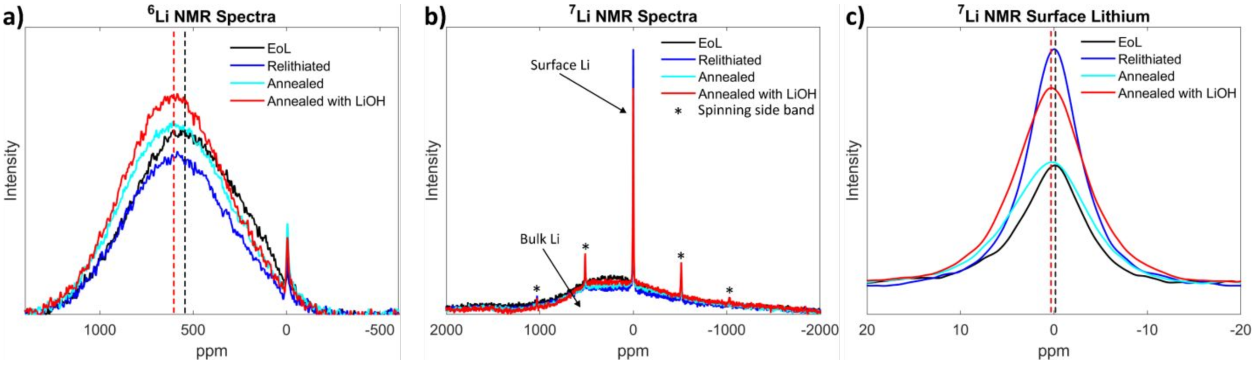

Figure S5: MAS-NMR results. a) ^6Li spectra, with EoL and annealed with LiOH peak locations identified. b) ^7Li spectra, showing bulk and surface lithium. b) ^7Li surface (diamagnetic) lithium, with EoL and annealed with LiOH peak locations identified.

Table S2: Relative amounts of bulk versus surface lithium, from ^7Li integrated peak areas.

Sample	Surface Li (%)	Bulk Li (%)
EoL (washed)	3.67	96.33
Relithiated	8.38	91.62
Annealed	4.65	95.35
Annealed with LiOH	4.91	95.09

The a-axis lattice parameter of the NMC layered oxide phase is shown in Figure S6a. It has been shown that as the nickel oxidation state changes from 3+ to 2+, the Ni-O bond increases in length in a layered oxide structure [7]. Thus, an increase in the a-axis of the layered oxide phase is related to the increase in Ni^{2+} in the structure, which correlates with the amount of cation mixing. Indeed, we see that the spike in the a-axis in Figure S6a of the relithiated + LiOH material corresponds to the spike in the cation mixing (Figure 4d) for that sample at the same temperature.

The ratios of Ni/Mn/Co were fixed to 0.65/0.20/0.15 for the RS phase, and the transition metal (TM) site also included lithium, using: $1 - \mathrm{Li_{TM}} = \mathrm{Ni_{TM}} + \mathrm{Mn_{TM}} + \mathrm{Co_{TM}}$. However, since lithium is essentially transparent to X-rays, the value $\mathrm{Li_{TM}}$ includes both lithium occupancy and vacancies. This can be seen in Figure S6b, where at room temperature the EoL RS phase has less "lithium" than the relithiated RS phases, even though the RS lattice is smaller for EoL (Figure 4e). Since a lithiated RS phase tends to contract with increasing lithium, likely most of the increase in "lithium" for the relithiated phases is vacancies [8,9,10]. Additionally, once lithiation of the RS phase surpasses ~30%, the structure becomes closer to the layered oxide phase, further confirming that the 45% "lithium" that is found in the unannealed relithiated RS phase is mostly vacancies instead of lithium [8,9,10]. In general, both relithiated samples start out with higher RS cation disorder (lithium occupancy or TM vacancies) than the EoL material, suggesting that the RS that forms after relithiation has a high amount of TM vacancies. This disorder is fixed at lower temperatures for the relithiated RS phases compared to the EoL RS phase.

The RS crystallite size was fit with the Gaussian crystallite peak broadening parameter in TOPAS, and the crystallite size is plotted in Figure S6c. Since the RS phase is mostly a surface layer (as confirmed by STEM, Figure 2 and Figure S1) the crystallite size is very small (< 80 nm). The relithiated RS crystallite sizes start out slightly larger than the EoL RS size, which correlates with a higher RS cation disorder for those materials. At temperatures correlating to an increase in the wt% of the RS phase (Figure 4a), the RS phases increase in crystallite size, indicating the growth of the surface disorder layer, which includes the RS phase. After cooling, the RS crystallite size returns to what it was before heating for both relithiated samples, but the EoL sample retains larger crystallites of RS. [11]

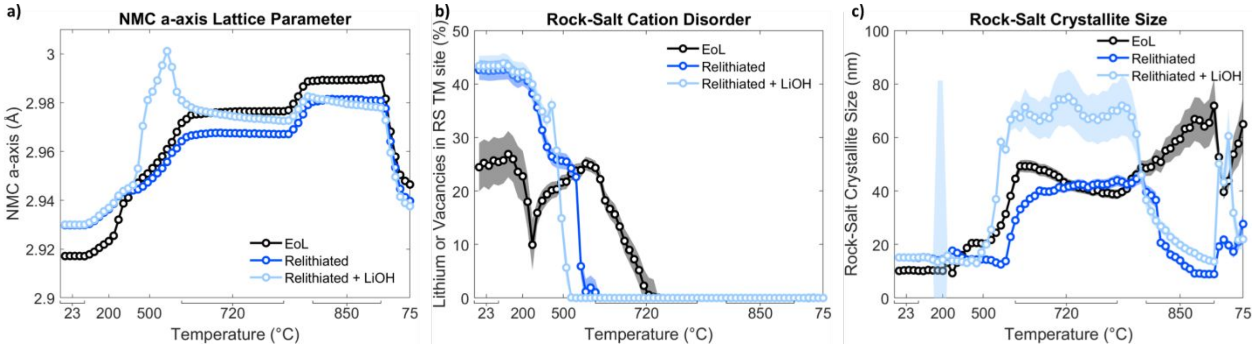

Figure S6: Additional parameters from Rietveld refinement. a) NMC a-axis lattice parameter. b) Amount of lithium or vacancies in the TM site in the RS phase. c) Crystallite size of the RS phase. Shaded regions indicate confidence intervals for parameters.

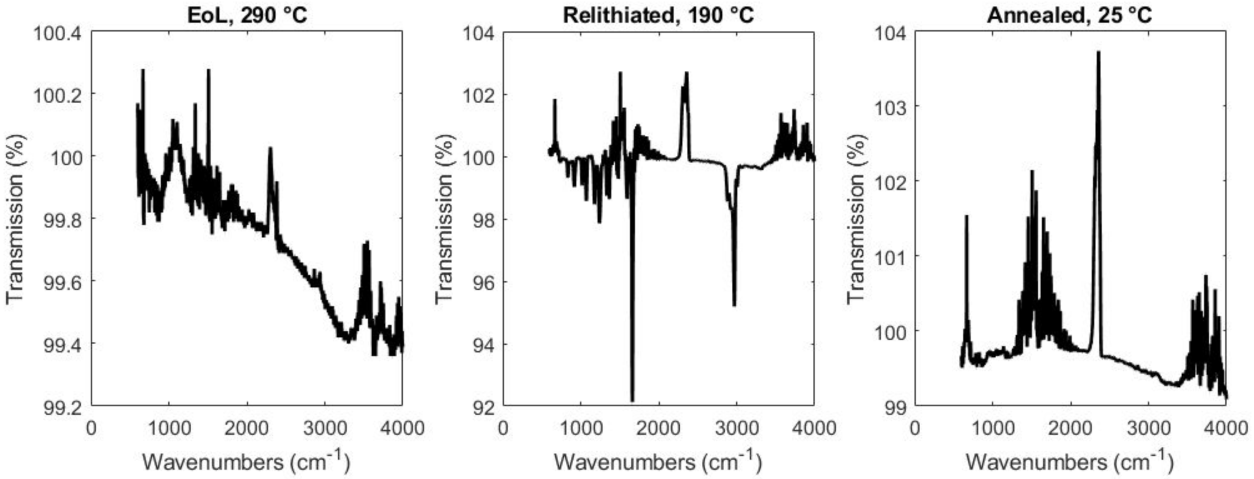

Figure S7: FTIR results from TGA-FTIR.

Fourier transform infrared spectroscopy (FTIR) was performed simultaneously with TGA measurements (Figure 5). The large mass loss at 190 ℃ for the relithiated sample is 2,5-di-tert-butyl-hydroquinone decomposition (as shown in the middle plot in Figure S7). The other samples (EoL and annealed) only show background noise, plus peaks for CO_2 and water, both of which are present in the experimental setup. It is possible that there is mass loss that is not IR active, or the concentration is too small to be visible.

Segmentation, analysis, and calculation of depth-dependent statistics from the XANES data followed a similar procedure reported previously in Allen et al. [12]. Ni XANES maps are fitted using reference samples of $LiNiO_2$ (for Ni^{3+}) and NiO (for Ni^{2+}). The K-edge for Ni shifts to higher energy values for Ni^{3+} than Ni^{2+} [13]. Figure S8a shows the fitted percentage of $LiNiO_2$ character for pristine, EoL, relithiated, and annealed samples. Figure S8b shows the Ni edge peak locations for the four samples. Fitted and peak location maps are in good agreement, meaning the fits to the reference materials are good. We also see the core-shell structure in the pristine material that we see in the EoL and recycled materials, suggesting that the center of particles naturally have more cation mixing (or Ni^{2+} content).

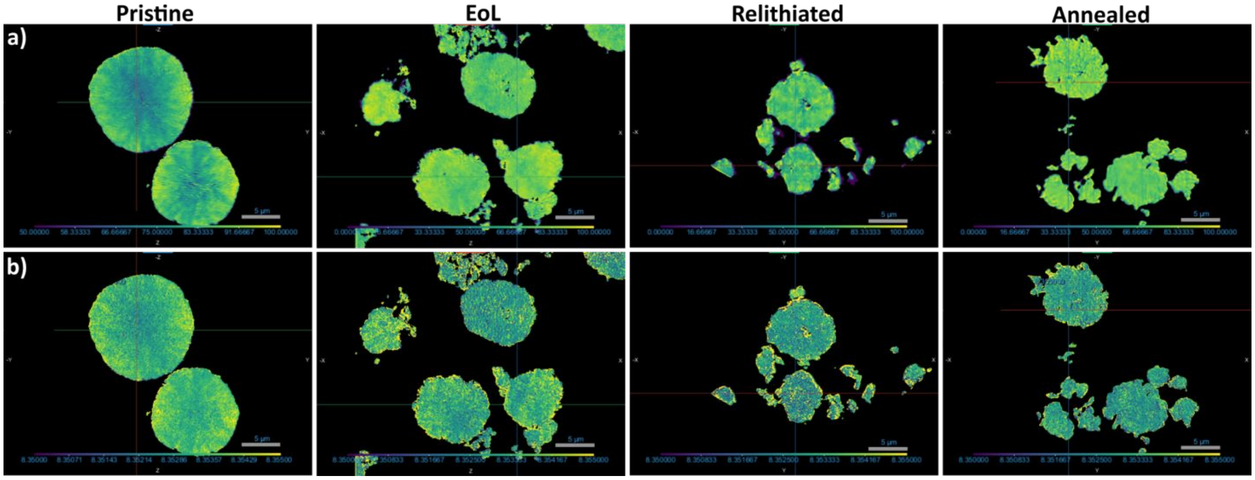

Figure S8: Comparison of % $LiNiO_2$ character to Ni edge peak position. a) % $LiNiO_2$ character (A high value correlates with more Ni^{3+}). b) Ni edge peak positions.

Particles were segmented and labeled (Figure S9a shows an example from the annealed sample). From this segmentation, particle surface is mapped through a surface mesh to calculate depth-dependent statistics, as shown in Figure 6. Depth-dependent statistics were calculated by choosing five secondary particles of similar size (Figure S9b), calculating their surfaces, and measuring the mean values as a function of depth from particle surface to core through Euclidean distance mapping (see Figure S9c for individual particle results for peak positions).

Particle volumes can also be calculated with this method, and averages of the depth-dependent statistics can be found for each particle. For example, Figure S9d shows the average percentage of $LiNiO_2$ character for each particle. For the EoL sample, smaller particles have more Ni^{2+} content (darker blue), suggesting that these particles have a higher cation mixing amount because they lost lithium inventory first before the larger particles. In both the relithiated and annealed samples, the smaller particles still have more Ni^{2+} content. However, the percentage of $LiNiO_2$ character varies less for the annealed sample, indicating that the annealing step has fixed some of the cation mixing. The variation in cation mixing with particle size indicates that control of secondary particle size is crucial to relithiation and annealing success.

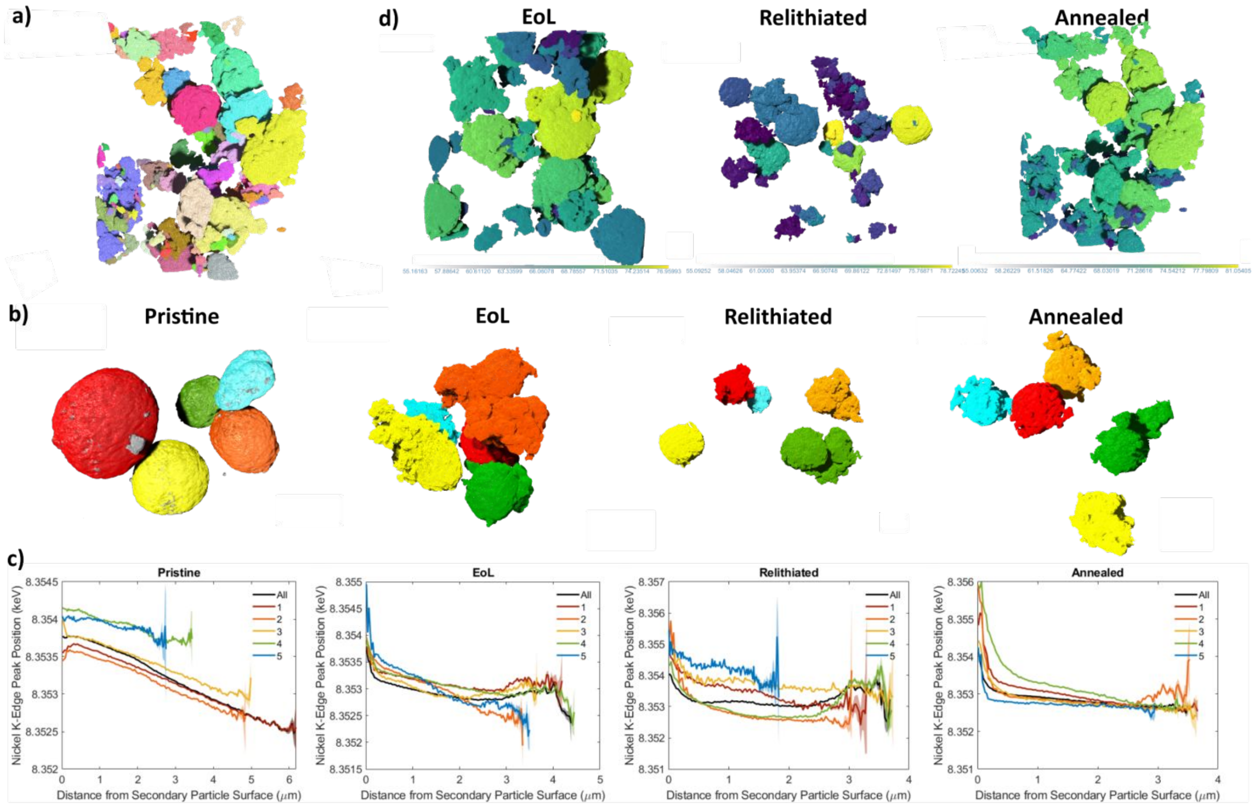

Figure S9: Additional images from XANES experiments. a) Individual particles segmented and labeled for the annealed sample. b) Chosen particles for depth-dependent statistics. c) Depth-dependent Ni K-edge peak positions for each particle. d) Average % $LiNiO_2$ character for each particle.

References

1. Fink, K.; Gasper, P.; Coyle, J. E.; Sunderlin, N.; Santhanagopalan, S. Impacts of Solvent Washing on the Electrochemical Remediation of Commercial End-Of-Life Cathodes. *ACS Applied Energy Materials*, **2020,** 3, 12212-12229.

2. Kobayashi, H.; Arachi, Y.; Kageyama, H.; Tatsumi, K. Structural determination of Li1-yNi0.5Mn0.5O2 (y ~ 0.5) using a combination of Rietveld analysis and the maximum entropy method. *Journal of Materials Chemistry*, **2004,** 14, 40-42.

3. Arachi, Y.; Kobayashi, H.; Emura, S.; Nakata, Y.; Tanaka, M.; Asai, T.; Sakaebe, H.; Tatsumi, K.; Kageyama, H. Li de-intercalation mechanism in LiNi0.5Mn0.5O2 cathode material for Li-ion batteries. *Solid State Ionics*, **2005,** 176, 895-903.

4. Cho, S.-J.; Chung, C.-C.; Podowitz-Thomas, S.; Jones, J. L. Understanding the lithium deficient LixNiyMnzCo1-y-zO2 (x < 1) cathode materials structure. *Materials Chemistry and Physics*, **2019,** 228, 32-36.

5. Ashton, T. E.; Baker, P. J.; Sotelo-Vazquez, C.; Footer, C. J. M.; Kojima, K. M.; Matsukawa, T.; Kamiyama, T.; Darr, J. A. Stoichiometrically driven disorder and local diffusion in NMC cathodes. *Journal of Materials Chemistry A*, **2021,** 9, 10477–10486.

6. Li, W.; Reimers, J. N.; Dahn, J. R. In situ X-ray diffraction and electrochemical studies of Li1-xNiO2. *Solid State Ionics*, **1993,** 67, 123-130.

7. Nishimura, Y. F.; Kondo, Y.; Oka, H. Estimation of the average oxidation number of nickel in a nickel oxide based on local structural information. *Journal of Power Sources*, **2020,** 446.

8. Goodenough, J. B.; Wickham, D. G.; Croft, W. J. Some Magnetic and Crystallographic Properies of the System Li+x Ni++(1-2x) Ni+++x O. *Journal of Physics and Chemistry of Solids*, **1958,** 5, 107-116.

9. Bianchini, M.; Roca-Ayats, M.; Hartmann, P.; Brezesinski, T.; Janek, J. There and Back Again—The Journey of LiNiO2 as a Cathode Active Material. *Angewandte Chemie International Edition*, **2019,** 58, 10434-10458.

10. Bianchini, M.; Schiele, A.; Schweidler, S.; Sicolo, S.; Fauth, F.; Suard, E.; Indris, S.; Mazilkin, A.; Nagel, P.; Schuppler, S.; Merz, M.; Hartmann, P.; Brezesinski, T.; Janek, J. From LiNiO2 to Li2NiO3: Synthesis, Structures and Electrochemical Mechanisms in Li-Rich Nickel Oxides. *Chemistry of Materials*, **2020,** 32, 9211-9227.

11. Kirwa, C. K.; Wang, E.; Son, S.-B.; Preimesberger, J. I.; Key, F. D.; Pupek, K.; Luo, H.; Keyser, M.; Coyle, J. Addressing Inherent Challenges to Chemical Relithiation of Cycled End-of-Life Cathode Materials. *Advanced Energy Materials*, **2025,** 2501809.

12. Allen, E.; Shin, Y.; Judge, W.; Wolfman, M.; Andrade, V. D.; Cologna, S. M.; Cabana, J. 3D Quantification of Elemental Gradients within Heterostructured Particles of Battery Cathodes. *ACS Energy Letters*, **2023,** 8, 1371-1378.

13. Abraham, D. P.; Twesten, R. D.; Balasubramanian, M.; Kropf, J.; Fischer, D.; McBreen, J.; Petrov, I.; Amine, K. Microscopy and Spectroscopy of Lithium Nickel Oxide-Based Particles Used in High Power Lithium-Ion Cells. *Journal of the Electrochemical Society*, **2003,** 150, A1450-A1456.
